# Principles of isomer stability in small clusters†

**DOI:** 10.1039/d2ma01088g

**Published:** 2023-02-28

**Authors:** Giuseppe Fisicaro, Bastian Schaefer, Jonas A. Finkler, Stefan Goedecker

**Affiliations:** a Consiglio Nazionale delle Ricerche, Istituto per la Microelettronica e Microsistemi (CNR-IMM), Z.I. VIII Strada 5 I-95121 Catania Italy Giuseppe.Fisicaro@imm.cnr.it; b Department of Physics, University of Basel, Klingelbergstrasse 82 CH-4056 Basel Switzerland

## Abstract

In this work we study isomers of several representative small clusters to find principles for their stability. Our conclusions about the principles underlying the structure of clusters are based on a huge database of 44 000 isomers generated for 58 different clusters on the density functional theory level by Minima Hopping. We explore the potential energy surface of small neutral, anionic and cationic isomers, moving left to right across the third period of the periodic table and varying the number of atoms *n* and the cluster charge state *q* (X^*q*^_*n*_, with X = {Na, Mg, Al, Si, Ge}, *q* = −1, 0, 1, 2). We use structural descriptors such as bond lengths and atomic coordination numbers, the surface to volume ratios and the shape factor as well as electronic descriptors such as shell filling and hardness to detect correlations with the stability of clusters. The isomers of metallic clusters are found to be structure seekers with a strong tendency to adopt compact shapes. However certain numbers of atoms can suppress the formation of nearly spherical metallic clusters. Small non-metallic clusters typically also do not adopt compact spherical shapes for their lowest energy structures. In both cases spherical jellium models are not any more applicable. Nevertheless for many structures, that frequently have a high degree of symmetry, the Kohn–Sham eigenvalues are bunched into shells and if the available electrons can completely fill such shells, a particularly stable structure can result. We call such a cluster whose shape gives rise to shells that can be completely filled by the number of available electrons an optimally matched cluster, since both the structure and the number of electrons must be special and match. In this way we can also explain the stability trends for covalent silicon and germanium cluster isomers, whose stability was previously explained by the presence of certain structural motifs. Thus we propose a unified framework to explain trends in the stability of isomers and to predict their structure for a wide range of small clusters.

## Abstract 2

We unveil the principles of isomer stability in small clusters. Our conclusions are based on a extensive statistical analysis of various structural and electronic descriptors on a huge database of isomers generated by ab-initio structure predictions.

## Graphical abstract

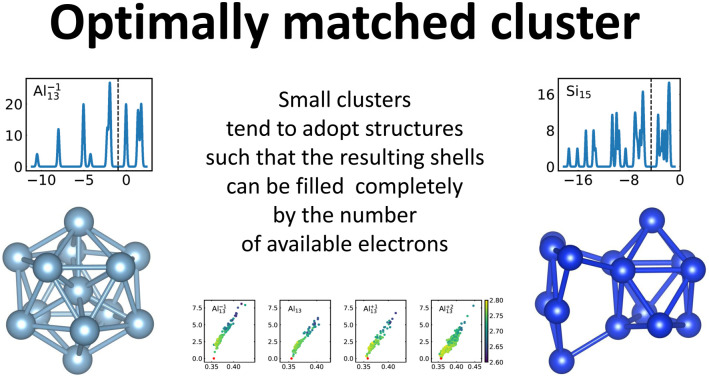

## I. Introduction

The physics of small clusters composed of a few to some hundred atoms represents an intriguing field of science. The physical and chemical properties of such clusters differ not only considerably from the properties of isolated atoms and bulk materials^1–4^ but can also vary considerably with the number of constituent atoms.^5–8^ The addition/removal of a single atom^5^ or a single electron^9^ may for instance lead to different ground state geometries. Different structures can then lead to completely different properties. Gold clusters that consist of a certain number of atoms can for instance exhibit catalytic activity.^10^ This high variability of cluster properties results in a lot of opportunities to create clusters with certain desired properties. Such clusters can then also be used as building blocks for cluster-assembled materials with certain desired properties.^11–18^ Fullerenes^19^ and their assembly in fullerides^20,21^ are a prominent example. Alkali doping of fullerides raises for instance the superconducting transition temperature of the cluster-based material by more than one order of magnitude.

Clusters can in principle be made of any number of atoms. The inspection of experimental mass spectra shows however that clusters of certain sizes can be found much more frequently than other sizes. For these sizes particularly low geometric ground states can be formed and they are called magic sizes. Numerous publications on the topic exist. We will extend the scope of these investigations by not only identifying geometric ground states as candidates for magic sizes, but by proposing general principles that also explain the relative stability of meta-stable isomers of clusters independently of whether they are magic sizes or not.

Two basic types of theories exist to explain the particular stability of clusters with magic sizes. The first theory is just based on purely geometrical considerations, while the second is based on the filling of electronic shells in a spherical jellium model.

For a simple pairwise potential icosahedral structures are highly stable. The smallest stable icosahedron can be formed by the 12 atoms located at the 12 corners of the icosahedron plus a central atom. The next two icosahedral structures then have one or two additional shells of atoms and consist of 55 and 147 atoms. So according to this geometrical theory the sizes containing 13, 55 and 147 atoms are predicted to be magic. Experimentally these sizes turn out to be magic sizes for rare gas atom clusters that are weakly interacting by van der Waals forces.^22,23^

The second theory establishes a connection between the number of valence electrons in the cluster and its stability. The concept of cluster electronic shells and the enhanced stability of clusters with completely filled shells starts from the work of Knight and co-workers on magic numbers in the mass spectra of free small sodium clusters.^24^ They detected a particular high stability for clusters containing 2, 8, 18, 20, 34, 40, 58… atoms. Knight and co-workers succeeded in rationalizing the experimental observations by means of the jellium model.^25–27^ The jellium model, an extension of the independent nuclear shell model,^27^ assumes that a gas of independent electrons is spatially confined by a model potential within a sphere. A uniform positive background charge mimics the ions in the cluster. The quantum mechanical energy levels of such a system together with their electronic occupations are given by |1s^2^|1p^6^|1d^10^|2s^2^|1f^14^|2p^6^|1g^18^|…, where s,p,d denote in the usual way the values *l* = 0, 1, 2 of the angular quantum number. The energetic ordering of the energy levels of electrons is however different from the one for a Coulombic potential. For most flat potentials^27^ the degenerate shells of jellium clusters can accommodate 2, 8, 18, 20, 34, 40, 58… electrons. Clusters with this number of valence electrons are considered *magic clusters* in this electronic theory.

For Na clusters it has been observed that for small sizes of up to 1500 atoms magic sizes are determined by electronic shell filling, whereas for larger sizes it is determined by complete geometrical shells.^28^ For the small clusters we are studying geometrical shell filling is in general not possible and only the Al^−1^_13_ and the Al_12_Si exhibit this effect since they have a completely filled icosahedral shell.

Various generalizations of the jellium model have been proposed that take into account deviations from spherical symmetry. Distortions of the spherical shape lead to a splitting of energy levels with different quantum numbers, *l*.^29^ For weak distortions, the splitting is however relatively small^30^ so that they still can be lumped together into the levels of a spherical potential. Many of the early works on non-spherical metal clusters^26,31,32^ were not based on systematic structure predictions at the density functional theory (DFT) level. Since only this approach can reliably find the ground state geometries, it is therefore questionable whether the assumed shapes and resulting properties were the correct ones. Distorted jellium models fail for instance to predict the icosahedral shape of the Na_55_ cluster that is obtained from DFT calculations.^33^ The jellium model and its generalizations work best for clusters made out of alkaline and alkaline earth metals. As soon as atomic p orbitals come into play, such as in Al clusters, a jellium like shell structure is absent in most cases.^34^ It should also be pointed out that the jellium model fails for transition metal clusters.^35^

Trends in cluster properties are related according to the jellium model to the occupation of molecular electronic shells in much the same way as the occupation of the atomic shells govern the properties of the elements in the periodic table. This suggests that it is possible to regard particular clusters as superatoms.^11,13,16,36–38^ The concept of magic clusters paves the way to superatoms physics. Following the findings on the halogen character of the Al_13_ cluster, various research groups, such as the experimental group of Castleman and Khanna's theory group^12,13,39^ explored the concept of superatoms. For instance, adding an electron to the 39 valence electrons of the neutral cluster leads to a fully occupied shell of the jellium model. This leads to a high electron affinity of 3.6 eV which is comparable to that of the chlorine atom. Therefore, the cluster behaves in a similar way to an halogen atom.^11,36,40,41^

We propose in this study a theoretical framework which goes beyond the jellium model. It is based on geometrical arrangements of the nuclei that lead to electronic shells that can be filled completely by the number of available electrons. These shells are however in general quite different from the shells predicted by the jellium model as well as its extensions. In contrast to any kind of jellium model we do not have to assume that the nuclei are completely smeared out into a uniform background charge, but we can use real point like nuclei. Our approach is also not restricted to explain the stability of magic size ground states, but can explain the stability of any cluster.

In order to substantiate our model we explore and analyse the potential energy surface (PES) of various neutral and charged cluster isomers at the density functional theory level. The clusters are made out of elements of the third period of the periodic table and span the groups I to IV. In this way we can compare the behaviour of metallic and covalently bonded clusters. To understand the general principles of cluster stability we do not only consider systems that are experimentally known to be magic sizes. Instead we study in general the influence of the variation of the number of atoms and electrons on low energy meta-stable isomers. We do not consider in this work transition metal clusters. So if we refer in the following to metals we mean simple metals.

## II. Methods

The minima hopping (MH) method^42^ was employed to systematically explore the potential energy surface of all clusters being studied. For every cluster several runs, starting from different initial structures, were performed. The runs were stopped when the clusters started to fragment frequently due to a high temperature of the molecular dynamics escape trajectories. Since the temperature is increased whenever a minimum is revisited, this indicates that a large fraction of all metastable structures has been found. Within MH, consecutive short molecular dynamics simulations are performed to escape from the local minima, followed by local geometry optimizations to efficiently sample the energy landscape. The initial velocities of the molecular dynamics escape trials are preferably aligned along low curvature modes of the local minima to exploit the Bell–Evans–Polanyi principle.^43,44^ A feedback mechanism based on a search history discourages revisiting already known local minima, allowing fast exploration of large portions of the PES. The transition states between the local minima on the PES were explored using the minima hopping guided path search with the stabilized quasi-Newton saddle optimizer.^45,46^

All structural searches were performed directly at the Kohn–Sham (KS) density functional theory level by coupling the MH method with the BigDFT package.^47–49^ BigDFT uses wavelets as basis functions, which are localized both in real and Fourier space and allows for an exact treatment of free boundary conditions without the need to introduce vacuum regions in the periodic dimensions for both neutral and charged systems. As a consequence, the code is well suited for the evaluation of the KS energies and forces for neutral and charged isolated clusters. Soft norm-conserving pseudo-potentials including non-linear core corrections^50,51^ were used. Except for Mg where we have used for backward compatibility^7^ the Local Density Approximation, the Perdew–Burke–Ernzerhof functional^52^ as implemented in the Libxc^53^ library was used. Spin polarized calculations were performed for all the clusters with an odd number of electrons. For a cluster with an even number of electrons, it was assumed that the electronic ground state is a singlet state, even though some exceptions to this rule are known.^54^ A zero electronic temperature was used for all the calculations, except for the Mg clusters with an odd number of electrons where a temperature of 3 × 10^−3^ Ha was used for faster convergence. All structures were relaxed using the Hellman–Feynman scheme until forces were less than 0.5 meV Å^−1^. We have made all data generated by this research, that are all structure coordinates of all minima found by the minima hopping method together with their structural and electronic properties, openly-available through the Materials Cloud Archive.^55^

## III. Results and discussion

Table 1 reports various structural and electronic properties of all global minima found by the MH method for all investigated systems. For the 58 different neutral and charged clusters listed in column 1 of Table 1 (we moved left to right across the third period of the periodic table varying the number of atoms *n* and the cluster charge state *q*, that is X^*q*^_*n*_, with X = {Na, Mg, Al, Si, Ge}, *q* = −1, 0, 1, 2), we found in total about 44 000 different minima on the potential energy surface of all these clusters. The global minimum (GM) gives the geometric ground state of the cluster whereas the local minima correspond to meta-stable structures.

**Table 1:** Structural and electronic properties of all global minima found for all neutral and charged clusters. The point group degeneracies are the symmetry-allowed orbital degeneracies^56^: 1 means that no symmetry related degenerate orbitals exist; an *n* > 1 means that an *n*-fold degenerate orbital can be present. The degeneracy of the calculated KS orbitals is also listed. Starred numbers indicate approximate degeneracies. Rows in bold highlight clusters with a magic number of electrons according to the jellium model. The nearest neighbor bond distances *τ*, extracted from the elemental crystalline ground state bulk structures,^57^ are given (in Å) by: *τ*_Na_: 3.633, *τ*_Mg_: 3.204, *τ*_Al_: 2.856, *τ*_Si_: 2.386, *τ*_Ge_: 2.495

Cluster	Number of electrons	Average bond distance [Å]	Shape factor *S*	Point group	Point group degeneracies	KS DFT degeneracies	Homo–Lumo gap [eV]	Chemical Hardness [eV]
Na^−1^_8_	9	3.547	0.36	*D*_2d_	[1, 2]	[1, 2]	0.40	1.25
**Na**_**8**_	**8**	**3.529**	**0.23**	***D***_**2d**_	**[1, 2]**	**[1, 2]**	**1.08**	**1.84**
Na^+1^_8_	7	3.592	−0.02	*C*_s_	[1]	[1]	0.48	1.67
Na^−1^_10_	11	3.560	0.46	*D*_2h_	[1]	[1, 4*]	0.33	1.23
Na_10_	10	3.571	0.51	*D*_2d_	[1, 2]	[1, 2]	0.60	1.51
Na^+1^_10_	9	3.587	0.36	*C*_2*v*_	[1]	[1]	0.34	1.53
Na_15_	15	3.616	−0.14	*C*_1_	[1]	[1, 2*, 4*]	0.31	1.25
Na^+1^_15_	14	3.615	−0.40	*D*_3_	[1, 2]	[1, 2, 4*]	0.79	1.54
Mg^−1^_10_	21	3.191	0.28	*D*_4d_	[1, 2]	[1, 2]	0.73	1.65
**Mg**_**10**_	**20**	**3.167**	**0.07**	***C***_**3v**_	**[1, 2]**	[1, 2, 3*, 5*]	**1.29**	**2.11**
Mg^+1^_10_	19	3.185	−0.17	*C*_3v_	[1, 2]	[1, 2, 3*]	0.98	1.71
**Mg** ^**+2**^_**10**_	**18**	**3.190**	**−0.22**	***C***_**3v**_	**[1, 2]**	[1, 2, 3*]	**0.89**	**2.06**
Mg^−1^_11_	23	3.192	0.30	*C*_s_	[1]	[1, 2*, 4*]	0.35	1.48
Mg_11_	22	3.172	0.35	*D*_3h_	[1, 2]	[1, 2, 4*]	1.32	2.07
Mg^+1^_11_	21	3.163	0.21	*C*_s_	[1]	[1, 2*, 3*, 4*]	0.33	1.73
**Mg** ^**+2**^_**11**_	**20**	**3.112**	**−0.07**	***D***_**3h**_	**[1, 2]**	[1, 2, 3*]	**1.53**	**2.30**
Mg^−1^_14_	29	3.193	0.30	*C*_2_	[1]	[1, 2*, 4*, 5*]	0.41	1.40
Mg_14_	28	3.107	−0.35	*C*_2_	[1]	[1, 2*, 3*, 5*]	0.57	1.66
Mg^+1^_14_	27	3.147	0.31	*C*_1_	[1]	[1, 2*, 3*]	0.28	1.54
Mg^+2^_14_	26	3.192	0.42	*C*_1_	[1]	[1, 2*, 3*]	0.84	1.87
Mg_15_	30	3.208	0.33	*D*_3h_	[1, 2]	[1, 2, 3*]	0.50	1.56
Mg^+1^_15_	29	3.208	0.32	*C*_2v_	[1]	[1, 2*, 3*]	0.56	1.53
Mg^+2^_15_	28	3.165	0.26	*C*_1_	[1]	[1, 2*, 3*]	0.46	1.66
Al^−1^_7_	22	2.715	0.38	*C*_3v_	[1, 2]	[1, 2, 3*]	0.74	2.05
Al_7_	21	2.711	0.28	*C*_3v_	[1, 2]	[1, 2, 3*]	0.88	2.25
**Al** ^+1^_7_	**20**	**2.735**	**0.06**	***C***_**3v**_	**[1, 2]**	[1, 2, 3*]	**1.96**	**2.86**
Al^+2^_7_	19	2.759	−0.06	*C*_2v_	[1]	[1, 2*, 3*]	0.44	2.26
Al^−1^_10_	31	2.742	0.14	*C*_s_	[1]	[1, 2*, 3*]	0.60	1.86
Al_10_	30	2.756	0.05	*C*_s_	[1]	[1, 2*, 3*]	0.59	1.97
Al^+1^_10_	29	2.771	−0.08	*C*_1_	[1]	[1, 2*, 3*, 4*]	0.55	2.01
Al^+2^_10_	28	2.746	0.67	*D*_2d_	[1, 2]	[1, 2, 3*]	1.26	2.38
**Al** ^**−1**^_**13**_	**40**	**2.756**	**0.00**	***I***_**h**_	**[1, 3, 4, 5]**	[1, 3, 4, 5, 10*]	**1.88**	**2.52**
Al_13_	39	2.771	−0.05	*D*_3d_	[1, 2]	[1, 2, 3*, 4*, 7*, 9*]	0.46	1.87
Al^+1^_13_	38	2.747	−0.16	*C*_s_	[1]	[1, 2*, 4*, 5*]	1.24	2.28
Al^+2^_13_	37	2.763	−0.16	*C*_s_	[1]	[1, 2*, 3*, 4*, 5*]	0.43	1.89
Al_12_Si^−1^	41	2.751	0.06	*D*_5d_	[1, 2]	[1, 2, 3*, 5*, 6*, 7*, 10*]	0.47	1.88
**Al_12_ Si**	**40**	**2.735**	**0.00**	***I***_**h**_	**[1, 3, 4, 5]**	[1, 3, 4, 5, 6*, 7*]	**2.00**	**2.73**
Al_12_Si^+1^	39	2.757	−0.04	*D*_3d_	[1, 2]	[1, 2, 3*, 5*, 9*, 10*]	0.49	1.96
Al_12_Si^+2^	38	2.739	−0.16	*C*_s_	[1]	[1, 2*, 5*, 7*]	1.30	2.33
Al_14_^−1^	43	2.766	0.22	*C*_s_	[1]	[1, 3*, 4*, 8*, 9*]	0.50	1.75
Al_14_	42	2.757	0.22	*C*_3v_	[1, 2]	[1, 2, 3*, 4*, 9*]	0.90	2.01
Al^+1^_14_	41	2.780	0.09	*C*_2v_	[1]	[1, 3*, 4*, 8*, 10*]	0.56	1.97
Al^+2^_14_	40	2.778	0.01	*C*_3v_	[1, 2]	[1, 2, 3*, 5*, 7*]	1.92	2.60
Al_15_	45	2.769	0.29	*C*_2*h*_	[1]	[1, 2*, 3*, 4*, 8*]	0.41	1.77
Al^+1^_15_	44	2.797	0.24	*D*_2h_	[1]	[1, 2*, 3*, 4*, 9*]	1.22	2.20
Al^+2^_15_	43	2.786	0.24	*C*_s_	[1]	[1, 2*, 3*, 4*, 6*, 7*]	0.48	1.85
**Si**_**10**_	**40**	**2.515**	**0.05**	***C***_**3v**_	**[1,2]**	[1,2,3*,7*]	**2.09**	**2.94**
Si^+1^_10_	39	2.530	−0.04	*C*_s_	[1]	[1, 2*, 3*, 4*, 5*]	0.70	2.31
Si^+2^_10_	38	2.541	−0.17	*C*_s_	[1]	[1, 2*, 4*, 5*]	1.55	2.76
Si_15_	60	2.528	0.31	*C*_3v_	[1, 2]	[1, 2, 3*, 6*]	2.19	2.79
Si^+1^_15_	59	2.529	0.29	*C*_s_	[1]	[1, 2*, 3*, 4*, 5*, 10*, 11*]	0.51	1.96
**Si** ^**+2**^_**15**_	**58**	**2.522**	**0.23**	***D***_**3h**_	**[1, 2]**	[1, 2, 5*]	**1.96**	**2.67**
Si^+3^_15_	57	2.512	0.81	*C*_2_	[1]	[1, 2*, 3*, 5*, 6*, 8*]	0.52	1.87
Si_20_	80	2.544	0.70	*C*_3v_	[1, 2]	[1, 2, 3*, 4*, 9*]	1.78	2.36
Si^+1^_20_	79	2.542	0.65	*C*_2v_	[1]	[1, 2*, 3*, 5*, 6*, 11*, 16*]	0.31	1.66
Ge_15_	60	2.742	0.33	*C*_s_	[1]	[1, 2*, 3*, 6*, 8*]	1.59	2.39
Ge^+1^_15_	59	2.739	0.31	*C*_2v_	[1]	[1, 2*, 4*, 5*, 8*, 12*]	0.47	1.89
**Ge** ^**+2**^_**15**_	**58**	**2.759**	**0.27**	***C***_**1**_	**[1]**	[1, 2*, 5*, 12*]	**1.20**	**2.25**

**Fig. 1:**
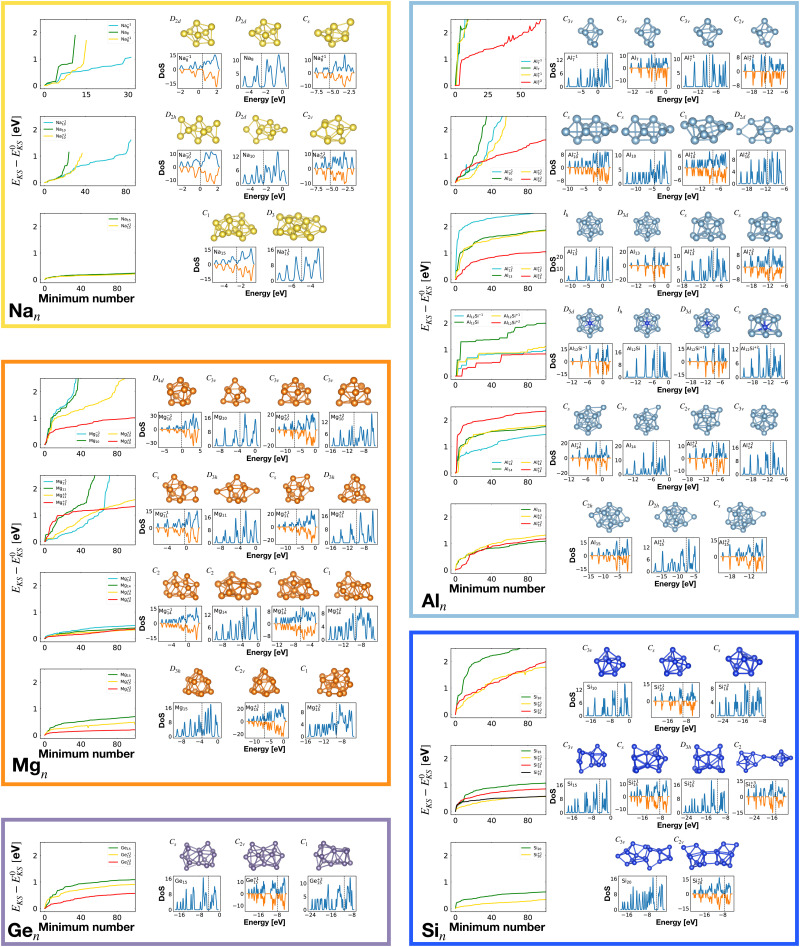
The structures of all global minima are shown together with their symmetry point groups and their electronic densities of states. The vertical dashed line on each global minimum DoS represents the Fermi level. The panels on the left of the structures show the KS energies *E*_KS_−*E*^0^_KS_ of the energetically sorted lowest 100 meta-stable structures with respect to the ground state, as a function of the sorting index for neutral and ionized clusters. If this function is rising rapidly, the energy increases strongly from one structure to the next energetically higher one.

Our global minimum structures for various cluster families are in agreement with previous structure prediction searches and experimental investigations. They are all shown in Fig. 1. The global minimum of Na_8_ agrees with previous Car–Parrinello simulations,^58^ the Na^−1^_10_ and Na_15_ GMs agree with the GM structures found by means of the CALYPSO structural search coupled to the simulated photoelectron spectra.^59^ Magnesium neutral global minima agree with the ones found by the CALYPSO structure prediction code^60^ and with other DFT studies.^61–63^ Global minima for neutral and charged aluminium clusters agree with previous DFT calculations for Al_7_.^64,65^ All the global minima of aluminum clusters match the ones determined by means of a multistage search approach (genetic algorithm, basin hopping, minima hopping and “by hands constructions”).^66–68^ Silicon global minima agree with the one determined by infrared multiple phonon dissociation spectra.^69^ Si_15_ GM corresponds to the one found by genetic algorithm explorations coupled to ion mobility measurements^70^ and the one determined by vibrational IR spectra coupled to DFT calculations.^71^ Si_20_ GM agrees with the one found by single-parent evolution search,^72^ the one determined by the “big bang” optimization method^73^ or with previous MH structure predictions.^9^

### A. Structural properties

To analyse the structural properties of the cluster isomers we have introduced several descriptors for the geometric shape of these clusters, namely the average coordination number and bond distance, the surface to volume ratio, the shape factor and the fingerprint distance to the global minimum.

#### 1. Average coordination number and bond distance

The coordination number *ζ*_*k*_ of an atom *k* equals the number of atoms within the first coordination shell. We define the first coordination shell by a soft cutoff that decreases continuously from one at the nearest neighbor distance *τ* to zero at the second nearest neighbor distance *ν*. These nearest neighbor and second nearest neighbor distances *τ* and *ν* were extracted from the elemental crystalline ground state bulk structures^57^ and are given (in Å) by: *τ*_Na_: 3.633, *τ*_Mg_: 3.204, *τ*_Al_: 2.856, *τ*_Si_: 2.386, *τ*_Ge_: 2.495 and *ν*_Na_: 4.195, *ν*_Mg_: 5.240, *ν*_Al_: 4.039, *ν*_Si_: 3.867, *ν*_Ge_: 4.075. The coordination number *ζ*_*k*_ for an atom *k* in a cluster is then given by1
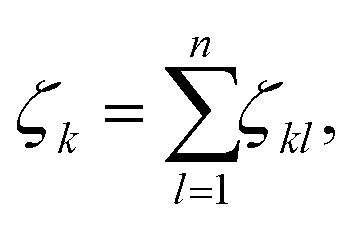
*n* being the total number of atoms in the cluster. The contribution *ζ*_*kl*_ of atom *l* is2
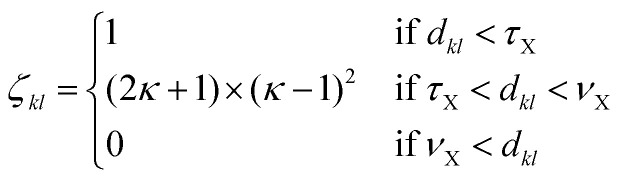
where *d*_*kl*_ is the distance between the *k* and *l* atoms, and *κ* = (*d*_*kl*_ − *τ*_X_)/(*ν*_X_ − *τ*_X_). By construction the *ζ*_*k*_ is identical to the number of nearest neighbors in the bulk structure.

The average coordination number *ζ* associated to a particular isomer is the average of *ζ*_*k*_ over all atoms *k*. The average bond distance is defined as the average over all atoms *k* of a cluster isomer of the average over all distances between the *k* atom and all the other atoms *l* used in the calculation of the coordination number *ζ*_*k*_.

**Fig. 2:**
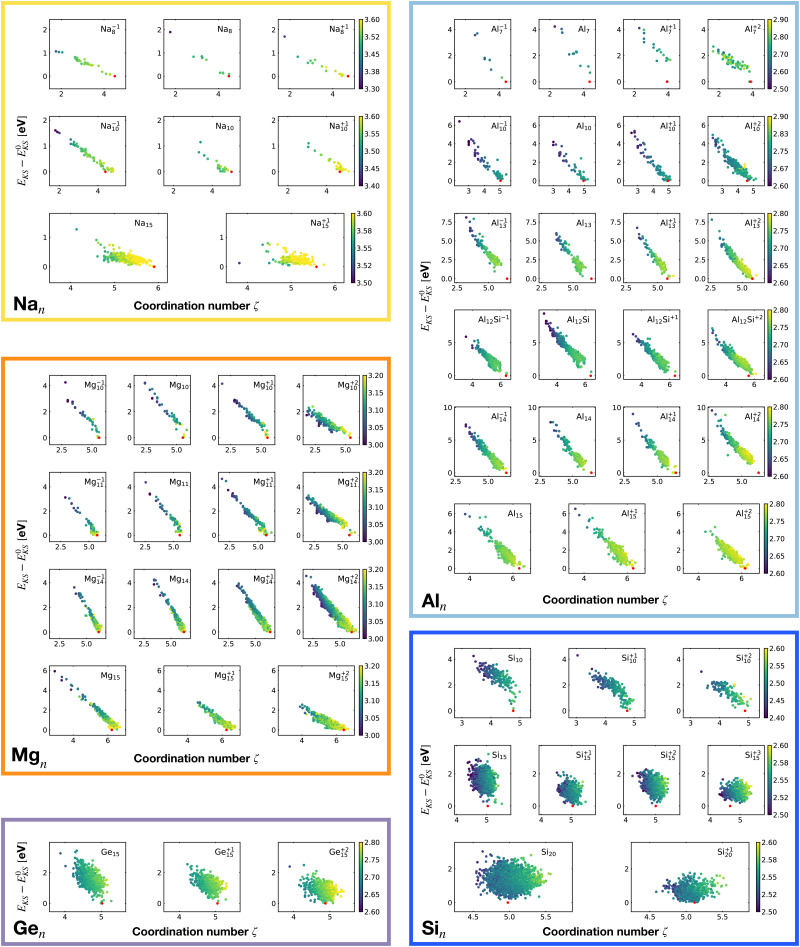
Correlation between the Kohn–Sham energy *E*_KS_ of isomers and their average coordination number *ζ*. Each sub-box belongs to a particular cluster family. *E*^0^_KS_ is the KS energy of the global minimum. The color scale for data dots is based on the average bond distance (Å). The red dot represents the global minimum for each PES.

Fig. 2 shows the correlation between the Kohn–Sham energy *E*_KS_ and the average coordination number *ζ*. Each sub-box is associated to a particular cluster family. The color scale for data dots indicates the average bond distance. The red dot represents the global minimum for each particular PES.

We notice a remarkable and nearly linear correlation between the Kohn–Sham energy *E*_KS_ of the isomers and their average coordination number *ζ* for all examined simple metals. The global minima of all the metallic systems are characterized by a high average coordination number *ζ* with a value higher than five or six depending on the cluster size *n*. Even though, as already pointed out, we do not study transition metal clusters, let us add for completeness that our findings for simple metals clusters are certainly not transferable to transition metal clusters. As is well known both pure^74^ as well as decorated^75^ small gold clusters have for instance planar shapes.

For the non-metallic clusters, where directional covalent bonds dominate, no such correlation can be seen. Like the corresponding bulk materials, the metallic clusters have much larger coordination numbers than the non-metallic clusters.

**Fig. 3:**
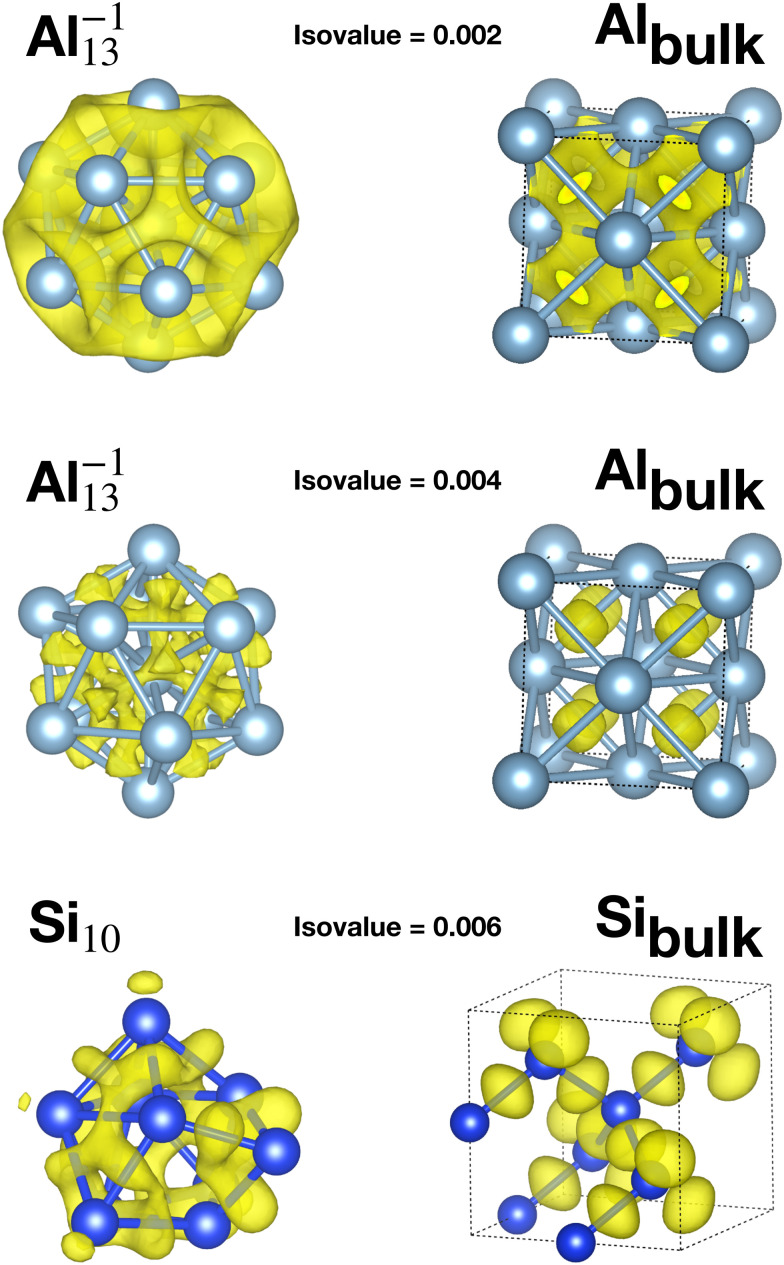
The difference between the selfconsistent DFT charge density and the sum of the atomic charge densities of spherically symmetrized atoms. For each isovalue only the positive part is shown which illustrates the flow of the electronic charge density when the structure is formed out of individual atoms. Top row shows the charge difference at an isovalue of 0.002 for Al^−1^_13_ and bulk fcc aluminum. The non-directional character of the bonding is clearly visible. In the panels of the second row a large isovalue of 0.004 was chosen to make the charge flow towards the center of the Al^−1^_13_ cluster visible. Bottom panels in the third row show the charge difference at an isovalue of 0.006 for the Si_10_ cluster and bulk silicon. The yellow blobs in the bulk silicon, representing the charge accumulation, are centered exactly in the middle of the bonds. This accumulation in the middle of all the bonds is not possible anymore in the cluster.

Table 1 reports the average bond distance of all global minima found in our minima hopping runs for neutral and charged clusters. In all cases the global minima of metallic clusters (sodium, magnesium and aluminium) have a shorter average bond length than the bulk phases. This is due to a redistribution of the charge from the region outside the surface of the cluster to the centers between neighboring atoms in the cluster as can be seen from the panels in the second row in Fig. 3, corresponding to an isovalue of 0.004. This charge redistribution is possible in a metallic system and leads to a lowering of the energy since the additional electronic charge inside the cluster is fully surrounded by the positively charged nuclei, whereas outside the surface it would only interact with a much smaller number of surface nuclei. A higher charge density in between neighboring atoms leads accordingly to the Hellmann–Feynman theorem to shorter bond lengths. This mechanism, which leads to bond shortening, is also encountered for the clusters of the other two elements that we examined, namely Na and Mg, as shown in Fig. S1 and S2 of the ESI.†

Apart from this charge flow towards bond centers, the bonding properties of the elemental metallic solid and the cluster are quite similar. As can be seen from the top panels in the first row of Fig. 3 (isovalue 0.002), there is a ring shaped electronic cloud around the atoms with a peak in the middle between nearest neighbors.

On the other side, the global minima of the covalent systems (silicon and germanium) are characterized by larger average bond distances compared with the corresponding bulk materials. Due to the strong directional bonding in insulators arising from hybridized orbitals, the charge in a disordered structure cannot flow into the bond region between two atoms to the same extent as in a solid with a perfect tetrahedral bonding pattern. This is also illustrated in the bottom panels, third row, of Fig. 3 (isovalue 0.006). In the ground state diamond structure of bulk silicon the sp^3^ hybridisation allows the charge to accumulate in the four tetrahedral positions which are exactly the bond centers. In the case of a Si cluster, the non-tedrahedral arrangement of the nearest neighbors does not allow for this kind of optimal charge accumulation in the middle between two atoms. Instead the charge is distributed in some kind of irregular way in the interstitial region. If there is less charge, in between two atoms, the bond length is longer as a consequence of the Hellmann Feynman theorem. In addition one can find core atoms that have a much higher coordination than the bulk atoms.

#### 2. Surface to volume ratio

Cutting surfaces costs energy because bonds are destroyed. Hence the surface area enters into many approximate formulas for the energy of nano-particles. For the small cluster sizes we are considering, the definition of the surface area is somewhat ambiguous. We will adopt in this study a widely used approach of calculating surface areas in the context of solvation,^76,77^ namely the soft-sphere implicit solvation model together with its calibration for an aqueous environment.^78,79^ The particular choice of solvent is irrelevant for our purposes. The dielectric cavity is based on analytic smooth spheres centered on each atom, and it is fully continuous from the vacuum-like inner regions to the external bulk solvent.

**Fig. 4:**
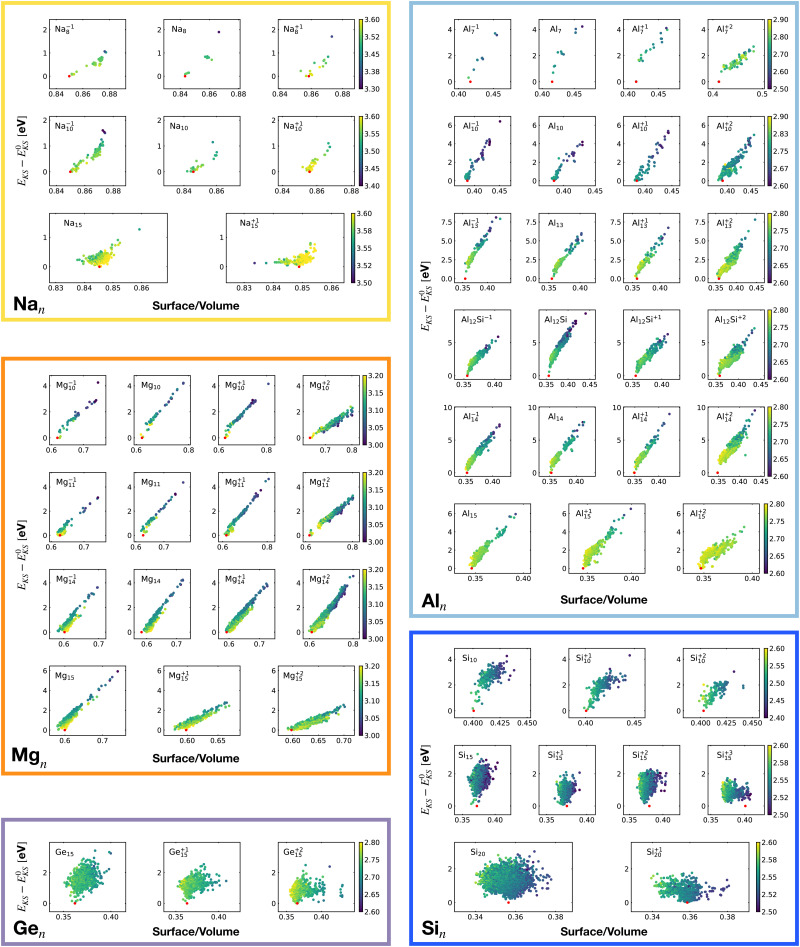
Correlation between the Kohn–Sham energy *E*_KS_ of isomers and the surface to volume ratio of their soft-sphere cavity. Each box belongs to a particular cluster family. *E*^0^_KS_ is the KS energy of the global minimum. The color scale for data dots is based on the average bond distance (Å). The red dot represents the global minimum for each PES.

For each isomer we computed the surface and volume of the associated soft-sphere cavity. Fig. 4 shows the correlation between the Kohn–Sham energy *E*_*KS*_ of the isomers and their surface to the volume ratio of their associated soft-sphere cavity. The plots show a high degree of correlation for all metallic clusters. In most cases the correlation is linear and low-lying isomers have a lower surface to volume ratio with respect to higher energy structures. The global minimum (red dots) coincides in general with the geometrical arrangement that minimizes this descriptor. Since the volume to surface ratio is strongly size dependent one can of course use this descriptor only for comparing clusters with the same number of atoms, where one can expect the volumes to be nearly constant.

So for a given number of atoms, there is a strong tendency to adopt compact shapes, but forming a nearly spherical cluster is not possible for any number of atoms as can be seen from the atomic arrangements shown in Fig. 1. The ground states show in general significant deviations from a perfect spherical shape. The icosahedron made out of 13 atoms (12 atoms from the 12 corners of the icoshedron and 1 central atom) is an exceptional case where one can form a quasi spherical shape. But with 15 spheres it is for instance not possible to obtain an approximately spherical shape by close packing spherical atoms of nearly identical size. The ground states of Na_15_ and Mg_15_ are consequently non-spherical.

The non-metallic clusters Ge_15_, Si_10_, Si_15_ and Si_20_ show hardly any correlation between the energy and the surface to volume ratio. The strong directional bonding prevents the cluster from adopting a nearly spherical shape.

#### 3. Shape factor

In addition to the sphere, oblate and prolate ellipsoids can also have a low surface to volume ratio. The eigenvalues of the inertia tensor of the cluster can describe these shapes. Denoting the eigenvalues in increasing order by *λ*_1_ ≤ *λ*_2_ ≤ *λ*_3_, we can define a shape factor *S* as

3 *S* = *λ*_2_/*λ*_3_ − *λ*_1_/*λ*_2_

**Fig. 5:**
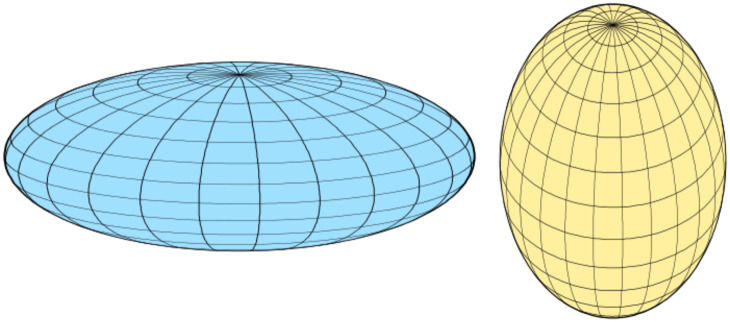
An oblate (left) and prolate (right) shape. Figure taken from: https://en.wikipedia.org/wiki/Spheroid.

The length of the structure along the eigenvectors of the inertia tensor, called the principal axes of inertia, is given by the reciprocal square root of the eigenvalues. For a strongly oblate structure, which has two long axes and a short one, we have *λ*_1_ ≈ *λ*_2_ ≪ *λ*_3_ and hence *S* ≈ −1. For a strongly prolate structure, which has one long axis and two short ones, we have *λ*_1_ ≪ *λ*_2_ ≈ *λ*_3_ and hence *S* ≈ 1. So *S* = 0 indicates a spherical shape whereas positive values of *S* indicate a prolate shape and negative values an oblate shape as shown in Fig. 5. The shape factor is actually not only zero for a sphere but for all Platonic solids (tetrahedron, cube, octahedron, dodecahedron and icosahedron). Such shapes are however not present in our data set, except the icosahedron which is indeed quite spherical.

**Fig. 6:**
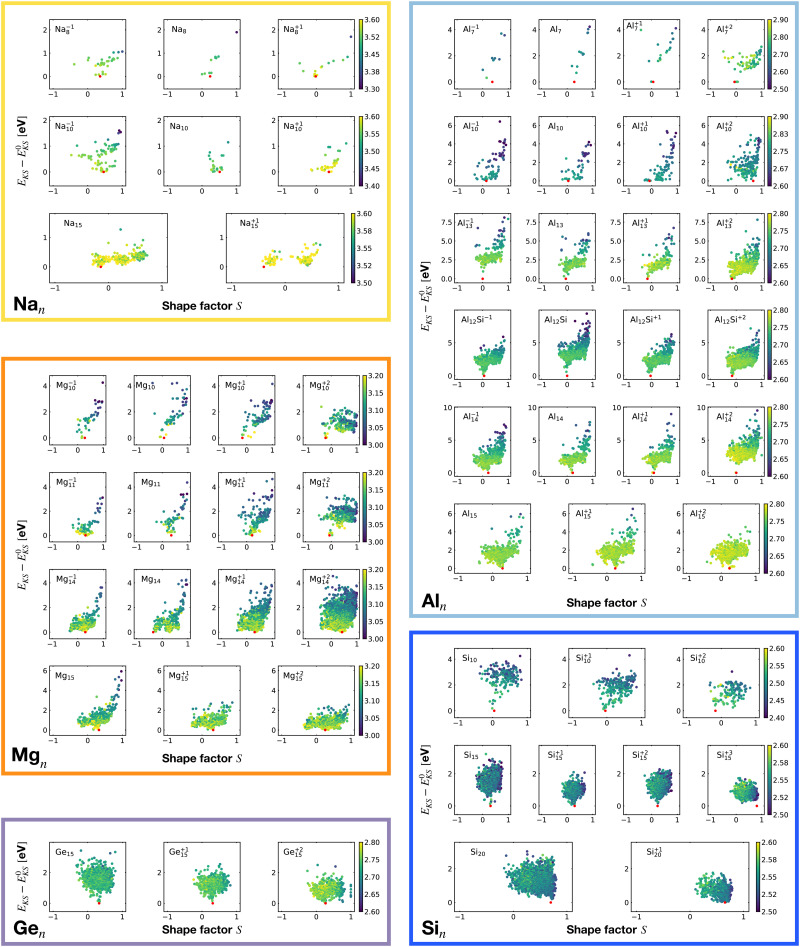
Correlation between the Kohn–Sham energy *E*_KS_ of isomers and their shape factor *S* defined by eqn (3). Each box belongs to a particular cluster family. *E*^0^_KS_ is the KS energy of the global minimum. The color scale for data dots is based on the average bond distance (Å). The red dot represents the global minimum for each PES.

Fig. 6 shows the correlation between the Kohn–Sham energy *E*_KS_ of a given cluster local minimum and its shape factor *S* defined by eqn (3). Values of the shape factor for all global minima are reported in Table 1. The preference for spherical shapes can be clearly seen for metallic clusters. Nevertheless many ground states, particularly with sizes in between the magic sizes of the jellium model, do not succeed to be close to spherical as can be deduced from the shape factors in Table 1. Higher energy metal cluster isomers typically adopt a prolate shape. While we limit our investigation to small clusters, a very similar behaviour based on geometrical shell completion was observed for a Na cluster with up to 147 atoms.^6^ For geometrically magic sizes of 55 and 147 atoms the ground state is a spherical icosahedron. For the sizes in between, disordered ground states were found that deviate significantly from a spherical shape. A non-spherical ground state was also reported for the Na_34_ cluster.^33^

No clear trends can be seen for the non-metalic clusters. For the Si_20_ which has a prolate ground state, the energy rises continuously when the shape becomes more spherical.

The principal axes of inertia are frequently aligned with the structure's symmetry axes. If a rigid body has an axis of symmetry of order *m*, meaning it is invariant under rotations of 360°/*m* about the given axis, this axis is a principal axis. When *m >* 2, the rigid body is a symmetrical top. If a rigid body has at least two symmetry axes that are not parallel or perpendicular to each other, it is a spherical top.^80^ The more symmetric the structure is, the more *S* tends toward zero.

It is worth noticing that low-energy minima of clusters with a magic number of electrons (or near magic number, that is ±1 electrons with respect to a magic number) always have an almost zero shape factor. This holds, in particular, for the potential energy surface of the magic clusters Na_8_, Mg_10_, Mg^+2^_10_, Mg^+2^_11_, Al^+1^_7_, Al^−1^_13_, Al_12_ Si, Al^+2^_14_, and Si_10_ (see Table 1). As a consequence, low-energy minima of magic clusters are highly symmetric and their arrangement tends toward a spherical top symmetry (see GM structures in Fig. 1). The spherical character of low lying isomers correlates with their higher coordination number *ζ* as shown in Fig. 2.

#### 4. Fingerprint distances to the global minimum

**Fig. 7:**
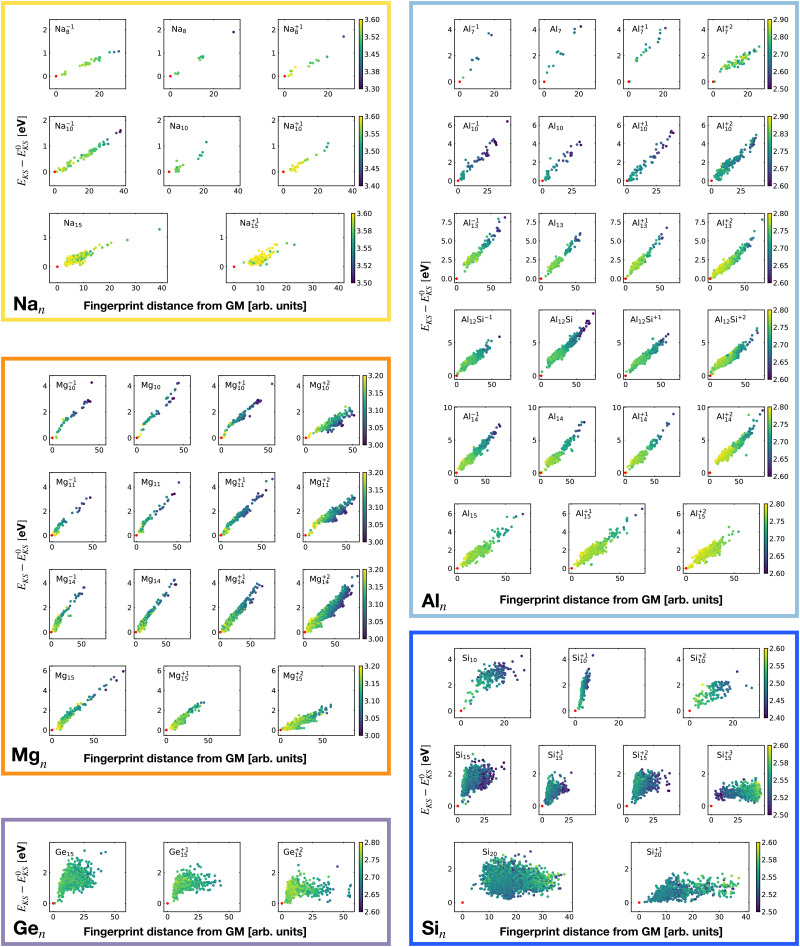
Correlation between the Kohn–Sham energy *E*_KS_ of isomers and their fingerprint distance from the global minimum. Each box belongs to a particular cluster family. *E*^KS^_0_ is the KS energy of the global minimum. The color scale for data dots is based on the average bond distance (Å). The red dot represents the global minimum for each PES.

**Fig. 8:**
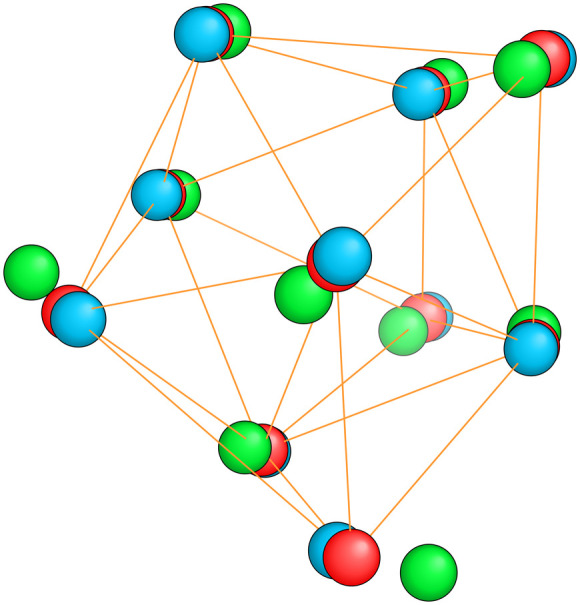
The red spheres show the saddle point structure of Mg_10_ that connects the ground state (blue spheres) with the first meta-stable structure (green spheres). To make the close similarity between these three structures visible, the radius had to be chosen much smaller than the standard covalent radius.

Fig. 7 shows the correlation between the Kohn–Sham energy *E*_KS_ of the isomers relative to their ground state and their fingerprint distance^81,82^ from the global minimum. A small fingerprint distance indicates a structural similarity whereas a large distance arises from important structural differences. We notice a remarkable linear correlation for all metallic clusters. This implies that whenever the structure becomes more similar to the ground state the energy will also go down. Hence there is a continuous driving force towards the ground state and the system will rapidly reach its ground state if the barriers along the way to the ground state are not too high compared to the thermal energy *k*_B_*T*. Even though we did not perform an exhaustive search for saddle points we found a few and they had very low barriers. For instance to cross from the ground state of Mg_10_ to the first meta-stable structure, barriers of only about a mHa have to be crossed. These low barriers are related to the fact that saddle points in these metallic systems are frequently sandwiched in between two closely related minima structures, as shown in Fig. 8. A similar but more extensive investigation of gold clusters^83^ has shown that most low energy structures can be transformed into other structures by crossing only very low barriers. Experimentally very low barriers have even been found for gold clusters of much larger size.^84^ Because of this combination of steady driving force towards the global minimum and low barriers these metallic systems are expected to be structure seekers and the ground state should therefore be reachable with moderate annealing.

Only a weak correlation between the energy and fingerprint distance can be seen for the non-metallic clusters. Extensive annealing might be required experimentally to find the ground state and in some cases it might actually not be reachable.^85^

### B. Electronic properties

In this section we will investigate which electronic properties lead to low energy cluster structures. The electronic system will be characterized in terms of the density of states, the matching of the KS DFT eigenvalue degeneracies with the number of valence electrons, the gap between the highest occupied molecular orbital (Homo) and lowest unoccupied molecular orbital (Lumo) and the chemical hardness.

#### 1. Symmetry and electronic shells

If a cluster belongs to a point group *G* because of certain symmetries, the Kohn–Sham Hamiltonian 
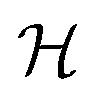
 commutes with the symmetry operators *R̂* of *G* if the self-consistent charge density has the same symmetry as the arrangement of the atoms in the cluster:4
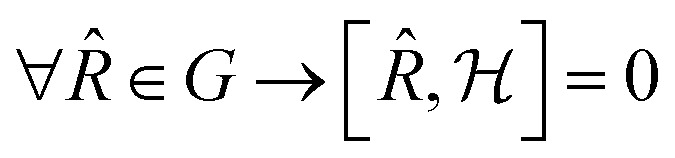


Consequently the Kohn–Sham orbitals are eigenfunctions of both *R̂* and 
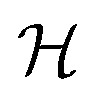
, and the eigenfunctions, that can be mapped onto each other by a symmetry operation, are degenerate. Filling an entire set of degenerate orbitals with the same number of possibly fractional electrons results in a charge density with the same symmetry as the cluster. Higher symmetries lead on average to higher degeneracies.

Table 1 gathers various structural and electronic properties of all global minima. Clusters that have a number of valence electrons that would classify the cluster size as magic in the jellium model are highlighted in bold in Table 1. However, as can be seen from the shape factors in Table 1, the shape of most of these clusters is quite non-spherical and the jellium model is not expected to be applicable. The failure of the jellium model can be seen from the fact that either the ideal shell ordering (1s, 1p, 1d, 2s, 1f, 2p) is reversed and/or some of these levels are split due to the symmetry breaking. These two effects have been studied in detail for Al clusters^34,86^ and we have also found them in many of our clusters as can be seen from their DoS (Fig. 1). Nevertheless, in virtually all cases completely filled shells stabilize the ground state of the clusters.

**Fig. 9:**
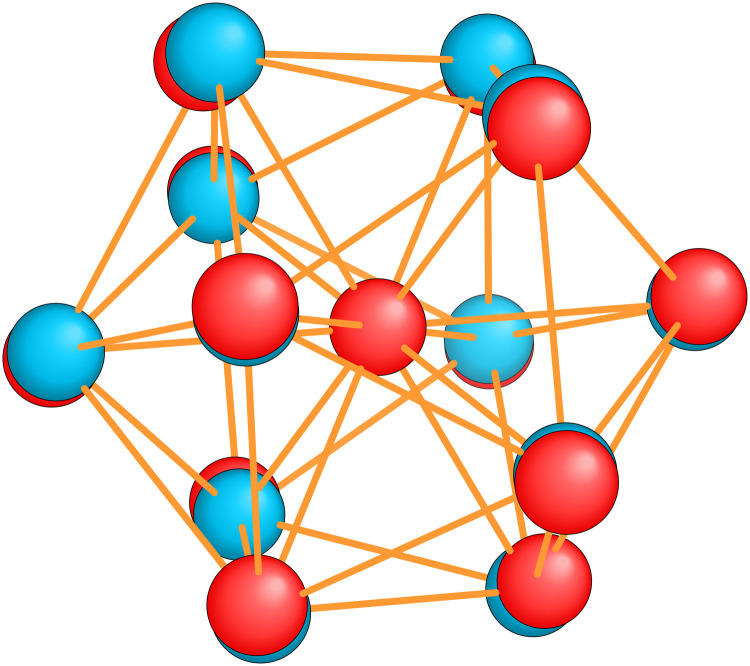
The structure of the icosahedral GM of Al^−1^_13_ (red spheres) and the slightly distorted Al_13_ ground state (blue spheres) with *D*_3d_ symmetry. The radius of the spheres had to be taken much smaller than the covalent radius to see the small distortion.

The shells are frequently related to exact degeneracies arising from symmetry, but approximate degeneracies that arise from small symmetry reducing Jahn–Teller distortions, are even more important. We considered KS eigenvalues to have an approximate degeneracy if they differ by less then 10^−2^ Ha. The Al^−1^_13_ cluster has for instance a high *I*_h_ symmetry. Removing one electron leads to a small relaxation (see Fig. 9) that reduces the symmetry to *D*_3d_. The exact 3 and 4-fold degeneracies that exist in the *I*_h_ group become approximate degeneracies (see Table 1) during the relaxation. The same effect can be seen in the non-metallic Si_15_ cluster. Removing one electron reduces the symmetry from *C*_3v_ to *C*_s_ and the exact two fold degeneracy becomes an approximate degeneracy. Several other similar cases can be deduced from Table 1.

We call this process, which allows for the complete filling of shells formed by exact or approximate degeneracies, optimal matching, because the degeneracies of the occupied eigenvalues have to match the number of available electrons. The eigenvalue spectrum is of course determined by the structure of the cluster. So in an optimally matched cluster the atoms have to be able to find positions such that the resulting spectrum has shells that can be filled completely by the available number of electrons. We can see from Table 1 that it is not possible to find such an optimal matching for any number of atoms and electrons, using only the degeneracies related to symmetry. It is therefore not true that the ground state of a cluster is necessarily of high symmetry. We will later on discuss some clusters where the highest symmetry structure is only a low energy meta-stable structure but not the ground state. The systems for which such an optimal matching is possible are however in the majority of cases of higher symmetry, which results frequently from a moderate distortion of an even higher symmetry structure as discussed above. Approximate degeneracies are particularly important for small clusters with few electrons where a single approximate degeneracy can already have a large stabilizing effect.

**Fig. 10:**
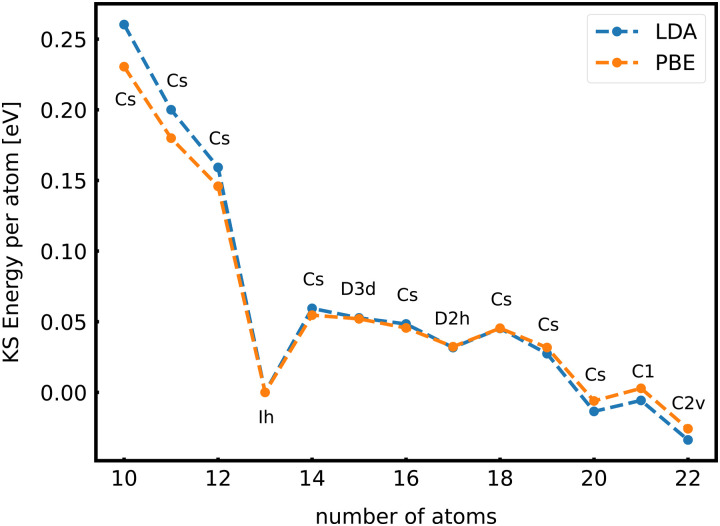
The energy per atom of anionic Al clusters relative to Al^−1^_13_ together with the point group of the cluster. The trends are independent of the exchange correlation functional used (LDA or PBE).

**Fig. 11:**
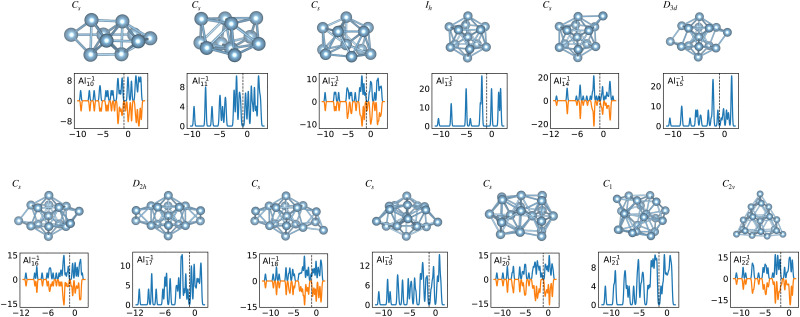
Structures and density of states of the series of anionic Al clusters. The high stability of Al^−1^_13_ relative to other cluster sizes, shown in Fig. 10, clearly coincides with the high degeneracies of this cluster. See also Fig. S3 of the ESI† for a similar figure augmented by the data used to make the figure plus selected structural and electronic properties.

When the optimal matching gives rise to a highly degenerate level, the cluster is particularly stable and is therefore expected to be found in large quantities in experimental spectra and to be thus a magic size. High degeneracy is in general related to high symmetry. The particularly low energy per atom of the ground state of optimally matched clusters is shown in Fig. 10 for the anionic Al clusters in the range between 10 and 22 atoms. This energy is on average of course decreasing as a function of cluster size and converging to the bulk value. Al^−1^_13_ has the highest symmetry (*I*_h_) and therefore by far the highest degree of degeneracy in the size range considered. As shown in Fig. 11, all the valence electrons are accommodated in seven shells arising from exact symmetries and in only five shells of near degeneracy. As a consequence it has the lowest energy per atom over a considerable range of cluster sizes.

**Fig. 12:**
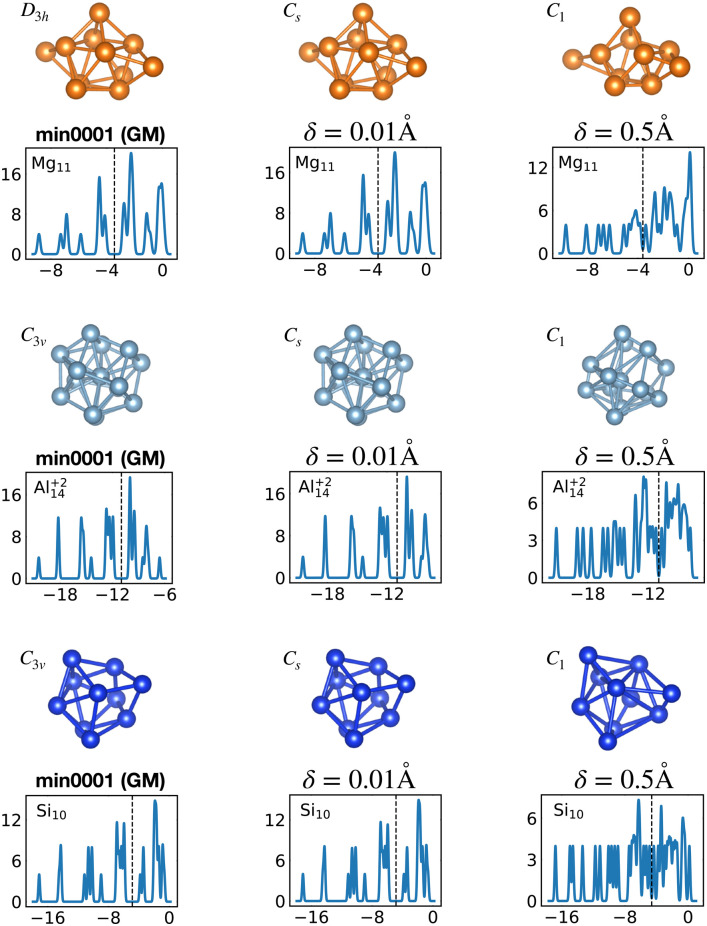
Lifting of the KS eigenvalue degeneracies by random atomic displacements away from the global minima. The effect for two different displacement amplitudes *δ* is shown, *δ* = 0.01 and 0.5 Å. See also Fig. S4 of the ESI† for a similar figure augmented by the data used to make the figure plus selected structural and electronic properties.

Fig. 12 shows the density of states for various perturbed and unperturbed global minimum configurations. We consider the GM of Mg_11_, with a non-magic number of 22 valence electrons, and the GM of the clusters Al^+2^_14_ and Si_10_, both with the magic number of 40 valence electrons. These GM are particularly stable as shown by the large energetic distance of the first meta-stable configurations relative to the ground state (sharp increase of *E*_KS_ in the relative leftmost panels of Fig. 1) and their large HOMO–LUMO gap, reported in Table 1. For each system we extracted the electronic properties for the unperturbed (*δ* = 0.0 Å) structure as well as for structures where each atomic coordinate has been randomly displaced with an amplitude *δ* between 0.0 and 0.5 Å. Since we obtained the DoS plots by a convolution with a Gaussian of width 0.1 eV, the lifting of the degeneracies becomes visible only for random displacements with an amplitude of 0.5 Å or more. This random perturbation also has the side effect of reducing the HOMO–LUMO gap (see Fig. S4 of the ESI†). This analysis elucidates the process of structural adjustment towards the global minimum driven by an optimal matching condition for the nuclei configuration and valence electrons.

**Fig. 13:**
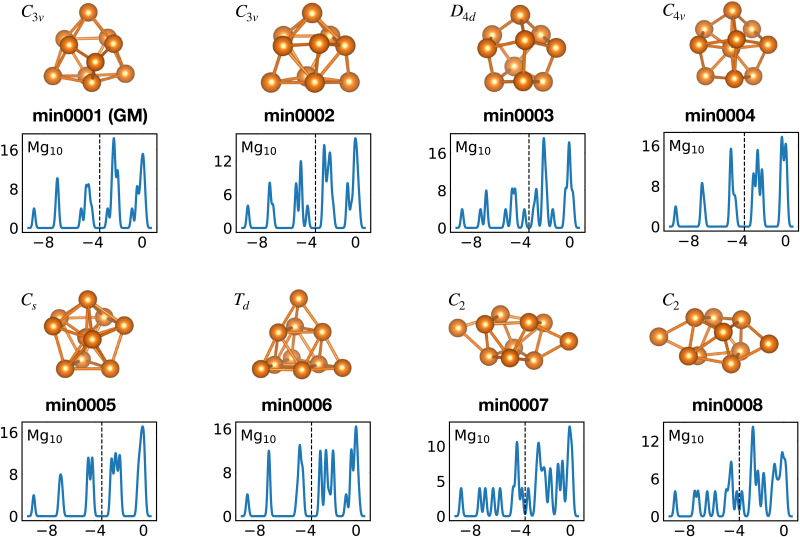
Structures and density of states of the first eight low-lying minima for the Mg_10_ cluster. See also Fig. S5 of the ESI† for a similar figure augmented by the data used to make the figure plus selected structural and electronic properties.

**Fig. 14:**
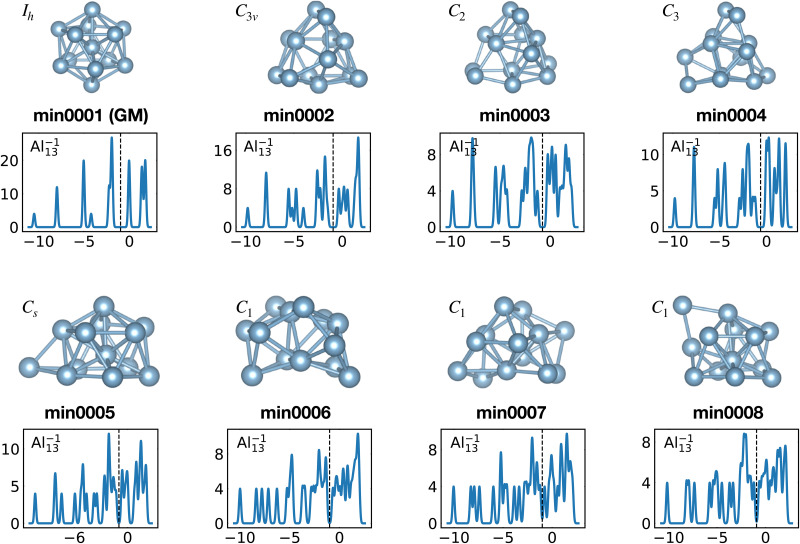
Structures and density of states of the first eight low-lying minima for the Al^−1^_13_ cluster. See also Fig. S6 of the ESI† for a similar figure augmented by the data used to make the figure plus selected structural and electronic properties.

**Fig. 15:**
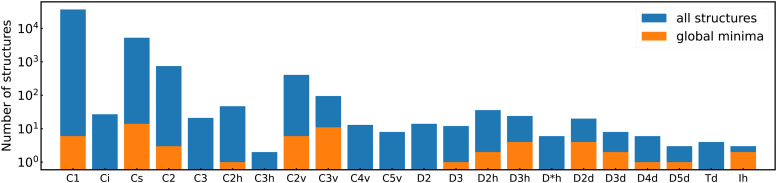
Histogram of the occurrences of all point groups in our cluster data set. Blue bins contain both ground states and meta-stable structures, and orange bins only ground state structures. Note the logarithmic scale along the *y*-axis. Point groups with a zero occurrence have been omitted.

Among the meta-stable structures high symmetry structures with filled shells can also be found frequently as detailed in Table 2. Fig. 13 and 14 show that all the low energy structures of these metallic clusters try to be spherical and to form completely filled shells (see also Fig. S5 and S6 of the ESI† reporting additional structural and electronic data for the same structures). Moving from the eighth minimum to the GM of Al^−1^_13_ in Fig. 14, the formation of electronic shells and the adoption of a spherical arrangement is clearly visible. However the high symmetry structures with completely filled shells get rarer with increasing energy of the isomers. As shown in the histogram of Fig. 15, the abundance of high symmetry structures in all meta-stable configurations is much lower than in the ground state structures. In the ground state structures the *C*_s_ and *C*_3v_ symmetry dominate whereas the overwhelming majority of the meta-stable structures have no symmetry, *i.e.* belong to *C*_1_.

**Table 2:** Some meta-stable configurations with completely filled shells. The degeneracies of the KS orbitals are listed left to right going from the lowest to the highest occupied Kohn–Sham eigenvalue

Cluster	Minimum	Number of electrons	Point group	Point group degeneracies	List of all KS DFT degeneracies
Mg_10_	1 (GM)	20	*C*_3v_	[1, 2]	[1, 1, 2, 1, 2, 2, 1]
	2	20	*C*_3v_	[1, 2]	[1, 2, 1, 2, 1, 2, 1]
	3	20	*D*_4d_	[1, 2]	[1, 1, 2, 1, 2, 2, 1]
	4	20	*C*_4v_	[1, 2]	[1, 2, 1, 1, 2, 1, 1, 1]
	6	20	*T*_d_	[1, 2, 3]	[1, 3, 1, 3, 2]
	12	20	*D*_2d_	[1, 2]	[1, 1, 2, 1, 2, 1, 1, 1]
Al^−1^_13_	1 (GM)	40	*I*_h_	[1, 3, 4, 5]	[1, 3, 5, 1, 3, 3, 4]
	2	40	*C*_3v_	[1, 2]	[1, 1, 2, 2, 1, 2, 1, 2, 1, 2, 1, 1, 2, 1]
	4	40	*C*_3_	[1, 2]	[1, 1, 2, 1, 2, 1, 2, 1, 2, 2, 1, 2, 1, 1]
	13	40	*C*_3v_	[1, 2]	[1, 1, 2, 1, 2, 2, 1, 1, 1, 2, 3*, 1, 2]
	105	40	*C*_3v_	[1, 2]	[1, 1, 1, 2, 2, 1, 2, 1, 2, 1, 2, 1, 2, 1]

The optimally matched clusters can also easily be recognized in the density of states plots in Fig. 1. In all these cases the density of states consists of a rather small number of narrow but high peaks arising from the degenerate shells and the Fermi level is in the middle of a large gap separating the fully occupied shells from the unoccupied shells. It does not matter whether the degeneracy comes from the spherically symmetric potential of the jellium model or whether it comes from another kind of high symmetry structure.

The energetically favorable high symmetry ground state structures always have several classes of equivalent atoms that see the same environment. In the Al^−1^_13_ cluster there are for instance two classes. The first comprises the 12 surface atoms of the icosahedron, and the second class consists only of the central atom. To obtain an overall low energy structure, the characteristic environments of the atoms defining the different classes must lead to low energy. When one transforms a high symmetry ground state into a meta-stable structure, at least one atom has to be moved. Since it was in a low energy position, the energy will typically increase strongly in such a move. In addition the corresponding moves of another atom in the same equivalence class will either give rise to an identical meta-stable structure or to a structure related by a symmetry operation. In both cases the increase in energy will be identical. Hence we can expect that we find only a relatively small number of structures in the funnel of a high symmetry ground state and that the energy gap between the ground state and the first meta-stable structure is relatively large. In a ground state with low symmetry all, or at least most atoms, have different environments, which are not all energetically optimal. Hence there are many possibilities to generate meta-stable structures and the energy increase will frequently be smaller since the starting point was already higher in energy. These expectations are confirmed by our data. The leftmost panel of each line in Fig. 1, corresponding to a cluster with a fixed number of atoms, shows the energy with respect to the ground state, *E*_KS_–*E*^0^_KS_, for the energetically sorted meta-stable structures. Since *E*_KS_–*E*^0^_KS_ increases rapidly for magic clusters, they have a deeper global minimum funnel containing a smaller number of meta-stable structures.

**Fig. 16:**
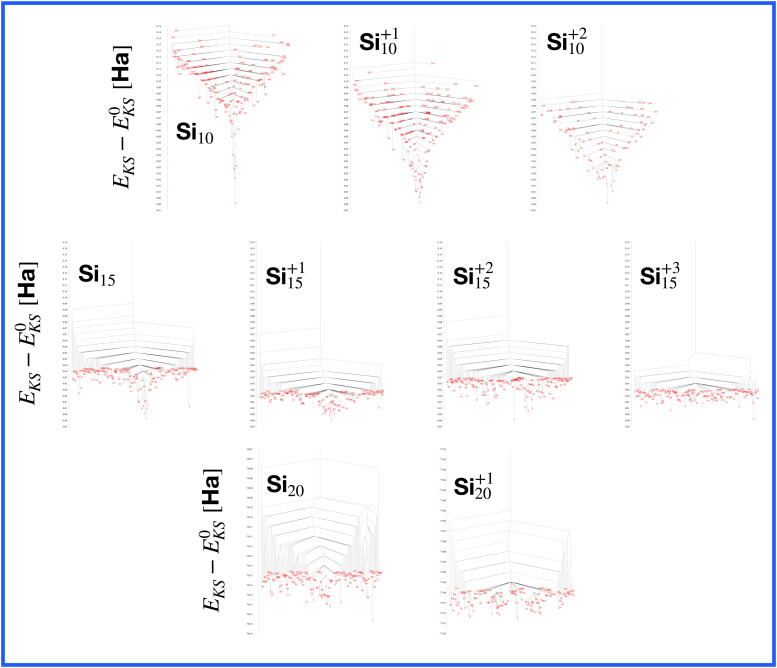
Disconnectivity graphs for selected silicon clusters.

A more detailed description of a funnel can be obtained from a disconnectivity graph which also provides the height of the barriers that have to be crossed when hopping from one minimum of the potential energy surface to another one. The disconnectivity graphs in Fig. 16 show, in agreement with the *E*_KS_ curves in the panels at the left of Fig. 1, that Si_10_ has the deepest funnel followed by the ionized Si_10_ clusters. In agreement with Fig. 7, the disconnectivity graphs also show that only Si_10_ and to a certain extent its ions are structure seekers whereas the other silicon clusters have a glassy energy landscape.

Since the spatial orbitals can always be filled with two electrons of opposite spin, a complete filling of the shells is only possible for clusters with an even number of electrons. For this reason all the magic clusters have an even number of electrons. If the neutral cluster has an odd number of electrons, the ionized cluster can sometimes be optimally matched. This is for instance the case in Al^−1^_13_ where the 40 valence electrons can completely fill all the shells of the jellium model. A complete filling of the shells is also observed for other clusters: the GM of Mg^+2^_11_ and Al^+1^_7_ with 20 electrons; the GM of Al_12_ Si and Al^+2^_14_ with 40 electrons.

For some well matched ground states, taking away or adding an electron can conserve up to slight distortions in the ground state such as in the Al_13_ cluster shown in Fig. 1 or 9. However in many cases the ground state configuration of the ionized cluster does not coincide with the ground state of the neutral cluster.^9^

#### 2. HOMO–LUMO gap and chemical hardness

**Fig. 17:**
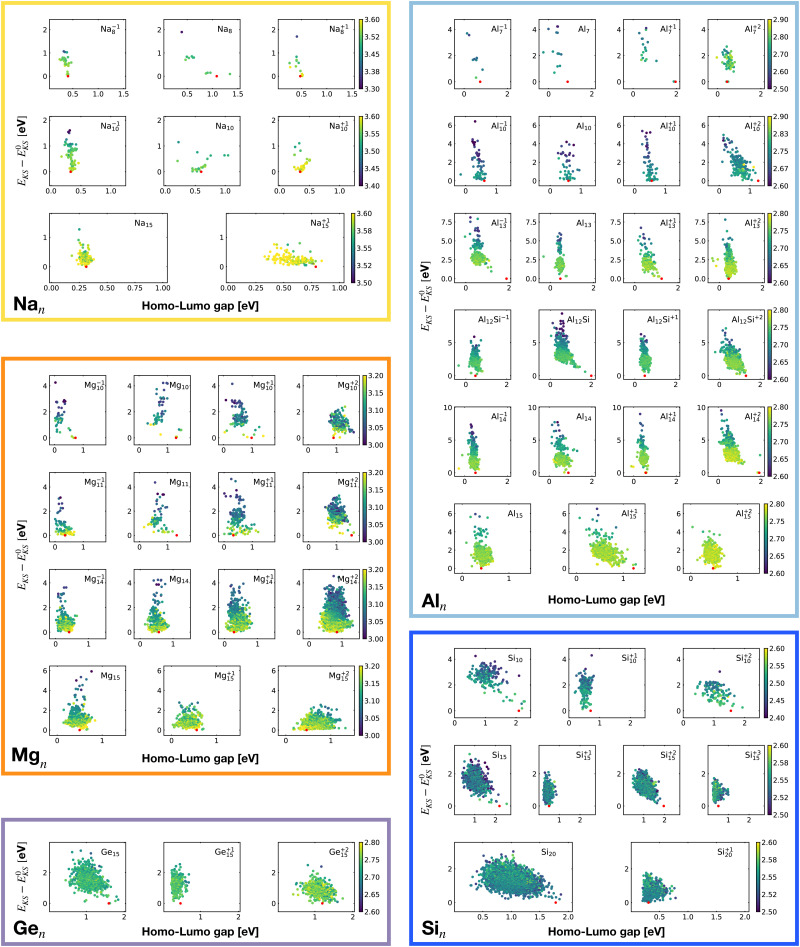
Correlation between the Kohn–Sham energy *E*_KS_ of isomers and their HOMO–LUMO gaps. Each box belongs to a particular cluster family. *E*^0^_KS_ is the KS energy of the global minimum. The color scale for data dots is based on the average bond distance (Å). The red dot represents the global minimum for each PES.

A large HOMO–LUMO gap is generally considered as an indication of a high cluster stability. Our data in Fig. 17 show no significant correlation between the HOMO–LUMO gap and the total KS energy. Comparing however different systems one sees that the HOMO–LUMO gap is particularly large for the magic systems (see Table 1). For the energetically unfavorable clusters with an odd number of electrons the gaps are particularly small.

Closely related to the HOMO–LUMO gap is the chemical hardness *η*, defined as5
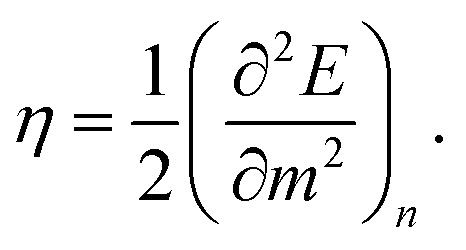


Applying a three-point finite difference approximation, we can extract the chemical hardness from the vertical ionization energy *E*^V^_*i*_ and the vertical electron affinity *E*^V^_*a*_6
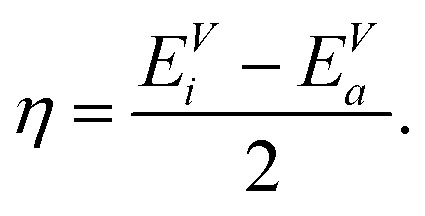


In a single particle scheme, the hardness is identical to the HOMO–LUMO gap. The vertical ionization energy *E*^V^_*i*_ is defined as the total energy difference between the (*m* − 1)-electron and the *m*-electron system with the cluster geometry kept fixed at the optimized *m*-electron configuration. Analogously, the vertical electron affinity *E*^V^_*a*_ is defined as the energy difference between the *m*-electron and the (*m* + 1)-electron system at the frozen *m*-electron configuration.

The maximum hardness principle^87^ was originally proposed by Pearson. It states that “at equilibrium, chemical systems are as hard as possible”. The chemical hardness quantifies the resistance to the changes in the number of electrons in the system, or to changes in the electronic density. A value of zero denotes maximum softness. For lithium clusters it was already shown that the magic size clusters are characterized by a particularly high hardness.^88^

**Fig. 18:**
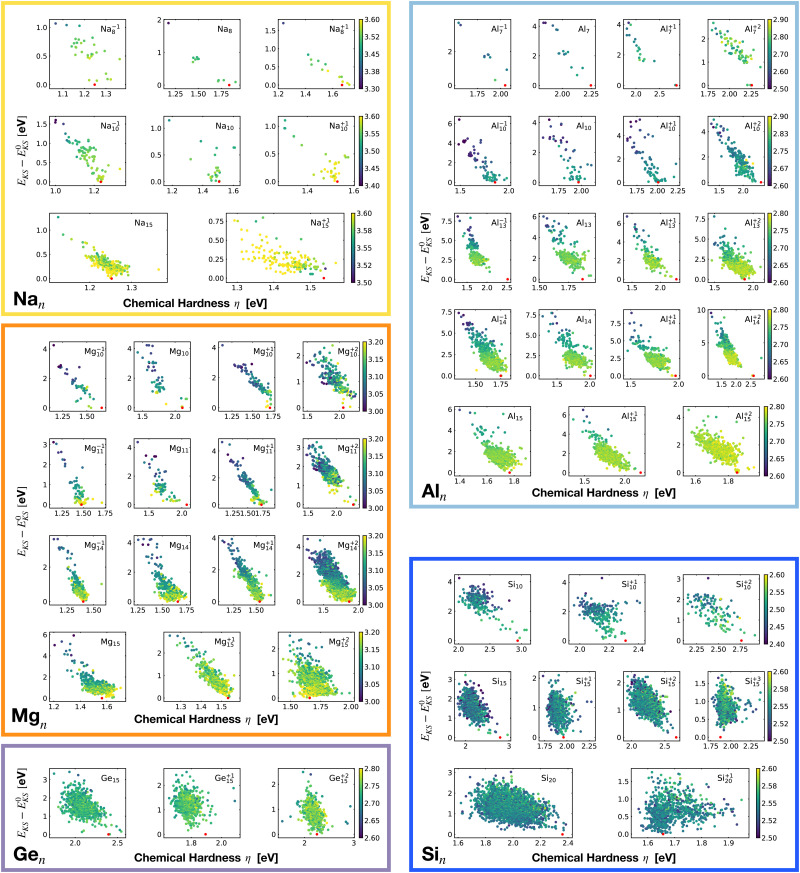
Correlation between the Kohn–Sham energy *E*_KS_ of isomers and their chemical hardness *η*. *E*^0^_KS_ is the KS energy of the global minimum. The color scale for data dots is based on the average bond distance (Å). The red dot represents the global minimum for each PES.

Even though the correlation of the total energy with neither the ionization energy nor the electron affinity is good, the correlation with the hardness is very good. As shown in Fig. 18 there is a clear linear correlation between the Kohn–Sham energy *E*_KS_ and the chemical hardness *η*. In most of the cases, the global minimum (red dot on each subgraph, data reported in Table 1) lies in the high *η* region of the PES. This is especially true for magic clusters or, in general, clusters with filled shells like the GM of Mg_11_, Si_10_ and Si_15_.

### C. Selected clusters

To illustrate our stability criteria, we will in the following discuss in more detail some selected clusters.

In its ground state, the Mg_11_ cluster can fill with its 22 electrons all the shells arising from a strongly non-spherical structure with *D*_3h_ symmetry. So optimal matching can explain this ground state, but since the structure is less compact than Mg clusters made out of a different number of atoms, it is not expected to be a magic size.

**Fig. 19:**
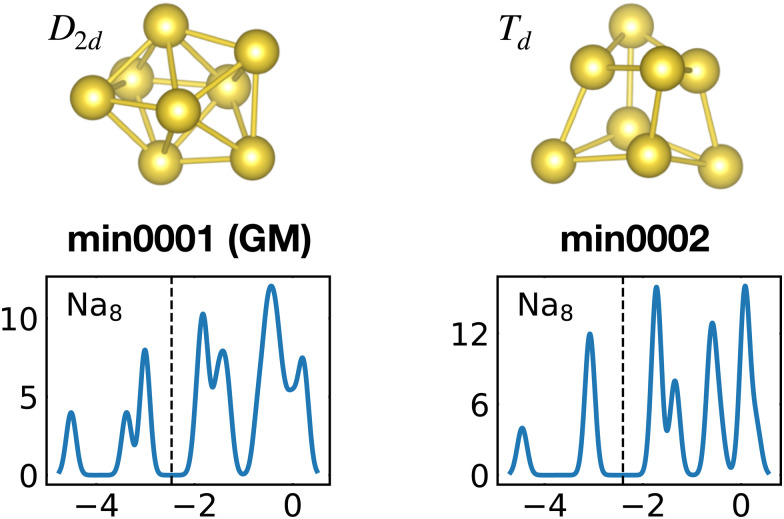
Structures and density of states of the first two low-lying minima for the Na_8_ cluster. The first meta-stable structure has higher degeneracies than the ground state, but is slightly more compact. See also Fig. S7 of the ESI† for a similar figure augmented by the data used to make the figure plus selected structural and electronic properties.

The competition between adopting a spherical shape and the complete filling of shells can be well observed in the Na_8_ and Mg_10_ clusters. The *T*_d_ structure of Na_8_ (Fig. 19 or Fig. S7 of the ESI† reporting additional structural and electronic data for the same structures), which allows for complete filling of the two shells, is only the first meta-stable structure, while the ground state has three filled shells. For Mg_10_ (Fig. 13) the highest symmetry structure is the tetrahedron which is however only the sixth meta-stable structure. The lower symmetry *C*_3v_ structure is the ground state. In both cases the ground states are slightly more compact than the more symmetric meta-stable structures.

**Fig. 20:**
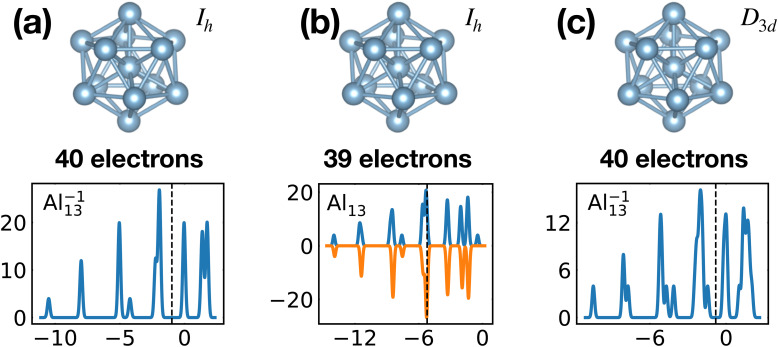
Density of states of 40 (a) and 39 (b) valence electrons, distributed over a perfect icosahedron with point group *I*_h_. This structure is the GM of Al^−1^_13_ and has point group degeneracies [1, 3, 4, 5]. (c) The DoS of 40 electrons distributed over the slightly distorted icosahedron *D*_3d_, GM of Al_13_, which has point group degeneracies [1, 2]. See also Fig. S8 of the ESI† for a similar figure augmented by the data used to make the figure.

Fig. 20 illustrates nicely the role of optimal matching in Al_13_. Starting from the Al^−1^_13_ cluster which is an optimally matched cluster, we can either take away an electron while freezing the *I*_h_ structure or keep the 40 electrons but transform the structure into the quite similar *D*_3d_ ground state structure of the neutral Al_13_. In both cases degeneracies are lifted. In the first case the reason is that the incomplete filling of the electronic shells leads to Hartree and exchange correlation potentials of lower symmetry. In the second case the ionic potential arising from the nuclei is of lower symmetry. Both processes reduce the stability of the cluster. See also Fig. S8 of the ESI† for a similar figure augmented by the data used to make the figure.

Optimal matching emerges also in the global minimum series of Al_14_ with charge states *q* = −1, 0, 1, 2. Analysing how GM structures and DoS (Fig. 1), as well as the shape factor *S* (Table 1), change with a varying number of electrons, the optimal match of 40 electrons allows for a more stable spherical arrangement of the 14 atoms.

The Al_12_Si cluster should be extremely stable according to all criteria. It has a nearly perfect spherical shape, because of its high symmetry multiple degenerate shells, that are completely filled, as well as a very high HOMO—LUMO gap and hardness. According to general belief, it should therefore not be reactive. However it turns out that this cluster reacts easily with a second identical cluster. It is therefore not possible to build a cluster assembled material based on this cluster.^89^ When two clusters are brought into contact during the assembly of such a material, they do not stay intact but form chemical bonds that destroy the 12 atom cage structure. A similar coalescence tendency was found for the Na_8_ cluster.^90^ For this magic cluster it was also shown that its spherical shape is destroyed upon deposition on a surface.

We analyzed the density of states of various clusters obtained from the global minimum of Al^−1^_13_, that is the icosahedron, replacing its central atom with elements of the IV and V groups: Al^−1^_13_, Al_12_ C, Al_12_ Si, Al_12_ Ge, Al_12_ Sn, Al_12_N^+1^, Al_12_P^+1^, Al_12_As^+1^, and Al_12_Sb^+1^. All of them hold the same magic number of valence electrons, that is 40, and, after their geometry optimization, the same icosahedron *I*_h_ symmetry of the Al^−1^_13_ global minimum is displayed. The KS DFT eigenvalue degeneracies are identical for all icosahedrons, and their DoS maintains an identical shell structure with respect to the one of the Al^−1^_13_ global minimum. For some icosahedrons the order of appearance for the KS eigenvalue degeneracies or the distances between groups of degenerate eigenvalues are slightly modified. For example, comparing the KS eigenvalue degeneracies [1, 3, 5, 1, 3, 3, 4] of Al^−1^_13_ with the [1, 3, 1, 5, 3, 4, 3] ones of Al_12_ C, we can notice an inversion of the third and fourth degeneracy levels. Similar differences appear when we compare other icosahedra. For all the Al_12_ X icosahedra, with X being one of elements investigated, exchanging the X central atom with a surface atom breaks the cluster symmetry and, as a consequence, reduces the KS DFT eigenvalue degeneracies. The structure is still meta-stable but the energy is considerably higher. These pieces of evidence can only be accounted for by the optimal matching description, not by a structure-less jellium model.

## IV. Conclusions

We have analysed the stability of a huge data set of isomer structures of representative clusters, containing the elements Na, Mg, Al, Si and Ge. The main criterion for stability is a perfect match between the degeneracies of the shells and the number of valence electrons that can fill these shells. These shells are frequently arising from approximate or accidental degeneracies. The atomic positions giving rise to a shell structure, that can accommodate the available number of electrons, can in general only be found by a structure search based on a global optimization algorithm. We call such a cluster whose shape gives rise to shells that can be completely filled by the number of available electrons an *optimally matched cluster*. If there are several optimally matched configurations the one which best satisfies other stability criteria will be lowest in energy. For our metallic structures we find a strong correlation between compactness and energetic stability. If the shape is nearly spherical the shell structure can be predicted by the jellium model if the electrons can be considered delocalized. Weak distortions away from a perfect spherical shape lead to splitting of the jellium shells and can change the energetic ordering of the shells. We have observed such small deviations from the ideal theoretical shell structures for most of our magic metallic clusters. Nevertheless the jellium model still correctly predicts magic sizes in these cases. If the cluster has a number of electrons that do not allow filling the shells of the jellium model completely, the model is of course not any more applicable. Our approach of optimally matched clusters is however still applicable. To obtain low energy, the cluster has to find an in general non-spherical shape that will give rise to shells that can completely be filled. So our approach can also explain the ground state structures of non-magic sizes.

For non-metallic clusters we could not find any tendency for spherical shapes. Hence jellium models are not applicable. As a matter of fact the stability of covalent clusters such as Si or Ge clusters was up to now nearly exclusively discussed in terms of certain structural motifs. Our approach of optimally matched clusters can however also be used in such cases and it can explain the in general strongly non-spherical ground state structures.

The bond lengths in metallic clusters are always considerably shorter than in the bulk metal. This is due to a flow of charge from the outside of the cluster to centers between neighboring atoms in the cluster. The effect is strongest for the low energy isomers.

In covalent clusters, the bond lengths are always longer than in the crystalline phase, since because of the directional bonding, it is not possible to accumulate charge exactly between each atom and all its nearest neighbors.

For all clusters the hardness and the energy difference between the first meta-stable structure and the ground state are good indicators for stability. In both cases large values indicate high stability. We have however no indications that a large hardness suppresses the chemical reactivity of clusters.

Our fingerprint-distance energy plots show that metallic clusters are structure seekers that can easily find their ground state by crossing only low barriers along a trajectory where they gain a significant amount of energy after each hop over a barrier. Experimentally they should therefore adopt their ground state with moderate annealing. This is however not true for covalent clusters. There the experimentally observed structure may not be the ground state but a structure that is kinetically more easily accessible.^85^

## Data availability

The data used to produce the results of this paper are openly available on the Materials Cloud Archive.^55^

## Conflicts of interest

There are no conflicts of interest to declare.

## Supplementary Material

MA-004-D2MA01088G-s001
