# Supplementary material for: Principles of isomer stability in small clusters
Source: Mater Adv. 2023 Feb 28;4(7):1746–68. doi: 10.1039/d2ma01088g (PMC10068428; doi:10.1039/d2ma01088g)
Supplement: MA-004-D2MA01088G-s001 [file MA-004-D2MA01088G-s001.pdf]

Electronic Supplementary Material (ESI) for Materials Advances.
This journal is © The Royal Society of Chemistry 2023

Supplementary information of:
Principles of isomer stability in small clusters

Giuseppe Fisicaro*

Consiglio Nazionale delle Ricerche, Istituto per la Microelettronica e Microsistemi (CNR-IMM), Z.I. VIII Strada 5, I-95121 Catania, Italy

Bastian Schaefer, Jonas A. Finkler, and Stefan Goedecker

Department of Physics, University of Basel, Klingelbergstrasse 82, CH-4056 Basel, Switzerland

In the following we show two additional analysis concerning the redistribution of the electronic charge density for selected Na and Mg clusters together with various figures from the main publication augmented by the data used to make the figure plus selected structural and electronic properties.

* Giuseppe.Fisicaro@imm.cnr.it

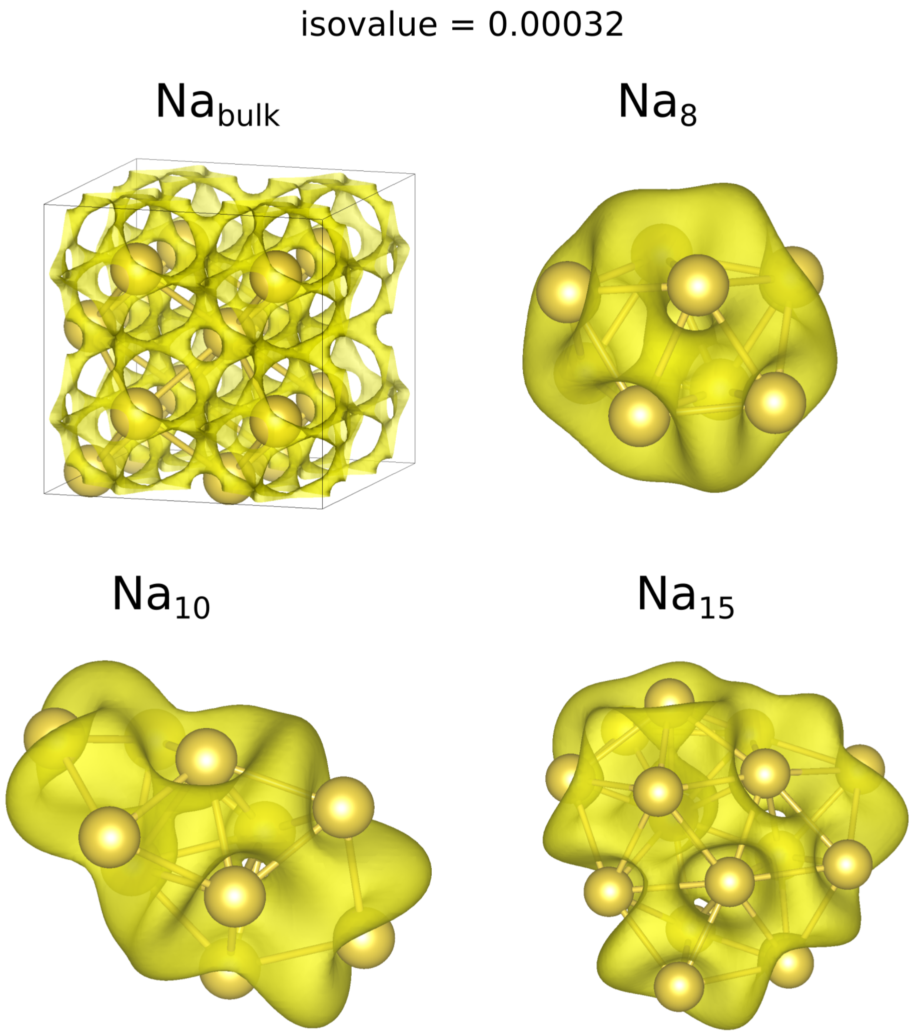

FIG. S1. Difference between the selfconsistent DFT charge density and the sum of the atomic charge densities of spherically symmetrized atoms. For each iso-value only the positive part is shown which illustrates the flow of the electronic charge density when the structure is formed out of individual atoms. All representations refer to an isovalue of 0.00032. Top row shows the charge difference for bulk Na and the global minimum of the Na_8 cluster. Bottom panels in the second row show the charge difference for the global minima of the Na_{10} and Na_{15} clusters. For large identical iso-charge values, the volume included by the iso-surface is always substantially larger in the case of the clusters compared to the solid. Since these regions of high charge densities are in between neighboring atoms, the bonds are contracted in the cluster compared to the solid.

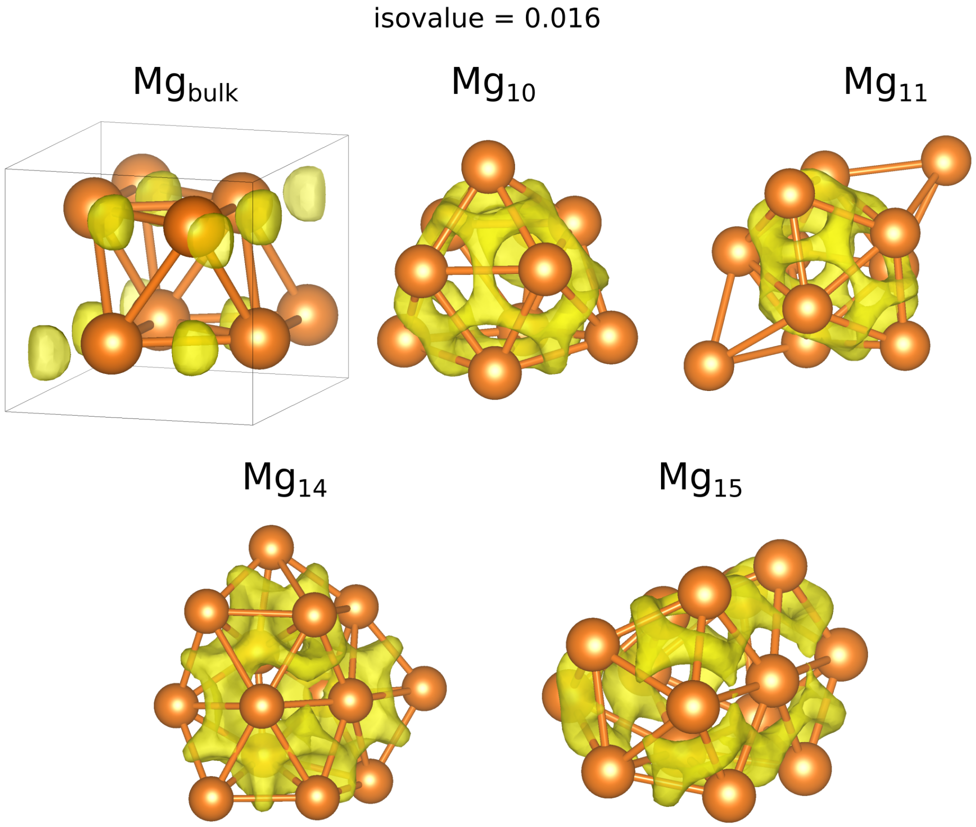

FIG. S2. Difference between the selfconsistent DFT charge density and the sum of the atomic charge densities of spherically symmetrized atoms. For each iso-value only the positive part is shown which illustrates the flow of the electronic charge density when the structure is formed out of individual atoms. All representations refer to an isovalue of 0.016. Top row shows the charge difference for bulk Mg and the global minima of the Mg_{10} and Mg_{11} clusters. Bottom panels in the second row show the charge difference for the global minima of the Mg_{14} and Mg_{15} clusters. For large identical iso-charge values, the volume included by the iso-surface is always substantially larger in the case of the clusters compared to the solid. Since these regions of high charge densities are in between neighboring atoms, the bonds are contracted in the cluster compared to the solid.

C_s

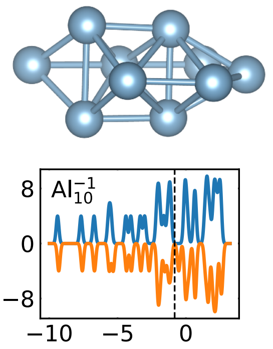

KS degeneracies within 2.72e-03 [eV]:
[[all ones], [all ones]]
KS degeneracies within 2.72e-01 [eV]:
[[1, 1, 1, 2, 1, 1, 1, 1, 3, 1, 3],
[1, 1, 1, 1, 1, 1, 1, 1, 1, 3, 1, 2]]

Homo-Lumo gap: 0.60 [eV]

KS eigenvalues e_i (e_i - e_{i-1}) [eV]
-9.39043
-7.67205 (1.71838)
-6.71987 (0.95218)
-5.64599 (1.07389)
-5.49185 (0.15413)
-4.38989 (1.10196)
-4.03225 (0.35764)
-3.34030 (0.69195)
-3.02046 (0.31984)
-2.12524 (0.89523)
-2.01982 (0.10541)
-1.92290 (0.09693)
-1.62185 (0.30104)
-1.32000 (0.30185)
-1.17704 (0.14296)
-1.12769 (0.04935)

C_s

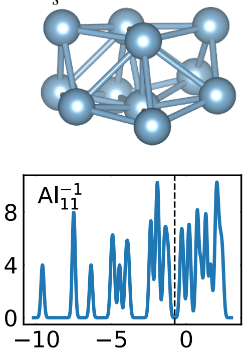

KS degeneracies within 2.72e-03 [eV]:
[all ones]
KS degeneracies within 2.72e-01 [eV]:
[1, 2, 1, 2, 1, 2, 2, 3, 3]

Homo-Lumo gap: 0.84 [eV]

KS eigenvalues e_i (e_i - e_{i-1}) [eV]
-9.59895
-7.51725 (2.08170)
-7.51063 (0.00662)
-6.35958 (1.15105)
-4.98933 (1.37025)
-4.85074 (0.13859)
-4.45487 (0.39587)
-4.01862 (0.43625)
-3.86174 (0.15688)
-2.40880 (1.45293)
-2.32608 (0.08273)
-2.01942 (0.30665)
-1.90858 (0.11085)
-1.88905 (0.01953)
-1.48527 (0.40379)
-1.35576 (0.12951)
-1.19663 (0.15912)

C_s

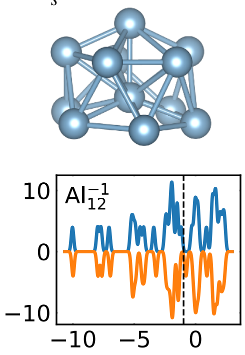

KS degeneracies within 2.72e-03 [eV]:
[[all ones], [all ones, 2]]
KS degeneracies within 2.72e-01 [eV]:
[[1, 2, 1, 3, 1, 1, 1, 6, 2, 1],
[1, 1, 1, 1, 3, 2, 1, 6, 2]]

Homo-Lumo gap: 0.39 [eV]

KS eigenvalues e_i (e_i - e_{i-1}) [eV]
-10.03622
-7.98412 (2.05210)
-7.71749 (0.26663)
-7.05398 (0.66351)
-5.25783 (1.79615)
-5.10507 (0.15276)
-4.91247 (0.19260)
-4.52256 (0.38991)
-4.22877 (0.29380)
-3.40871 (0.82006)
-2.47070 (0.93800)
-2.32554 (0.14516)
-2.09554 (0.23001)
-1.94908 (0.14646)
-1.94451 (0.00457)
-1.83207 (0.11245)
-1.51370 (0.31837)
-1.49140 (0.02230)
-1.17200 (0.31940)

I_h

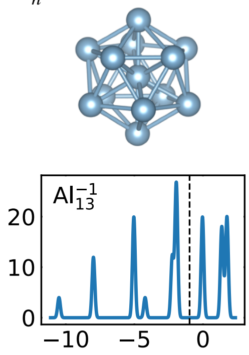

KS degeneracies within
2.72e-03 [eV]:
[1, 3, 5, 1, 3, 3, 4]
KS degeneracies within
2.72e-01 [eV]:
[1, 3, 5, 1, 10]

Homo-Lumo gap: 1.88 [eV]

KS eigenvalues e_i (e_i - e_{i-1}) [eV]
-10.49679
-7.98559 (2.51120)
-7.98559 (2.44908e-06)
-7.98558 (7.10716e-06)
-5.03997 (2.94561)
-5.03997 (3.91845e-06)
-5.03996 (9.17640e-06)
-5.03995 (1.23687e-05)
-5.03994 (7.31069e-06)
-4.21843 (0.82151)
-2.24053 (1.97791)
-2.24052 (2.63369e-06)
-2.24052 (6.30607e-06)
-1.97855 (0.26197)
-1.97855 (5.72271e-06)
-1.97854 (5.96459e-06)
-1.91787 (0.06067)
-1.91786 (1.14980e-05)
-1.91786 (5.82598e-06)
-1.91785 (6.71484e-06)

Figure continued on next page ...

C_s

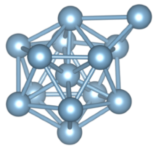

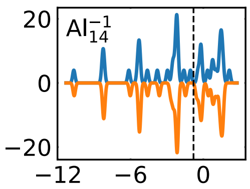

KS degeneracies within 2.72e-03 [eV]:
[[all ones, 2, 1, 1, 1], [all ones]]
KS degeneracies within 2.72e-01 [eV]:
[[1, 3, 1, 4, 1, 1, 1, 8, 1, 1],
[1, 3, 1, 4, 1, 1, 9, 1]]

Homo-Lumo gap: 0.50 [eV]

KS eigenvalues e_i (e_i - e_{i-1}) [eV]
-10.68338
-8.31731 (2.36607)
-8.25992 (0.05739)
-8.20007 (0.05985)
-6.14334 (2.05673)
-5.41821 (0.72513)
-5.30544 (0.11276)
-5.28567 (0.01978)
-5.24820 (0.03747)
-4.61258 (0.63562)
-3.79152 (0.82106)
-2.95343 (0.83809)
-2.54245 (0.41098)
-2.44144 (0.10101)
-2.27839 (0.16305)
-2.21293 (0.06546)
-2.20017 (0.01276)
-2.17036 (0.02981)
-2.17022 (0.00014)
-2.10456 (0.06566)
-1.60008 (0.50448)
-1.05885 (0.54123)

D_{3d}

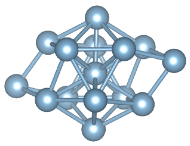

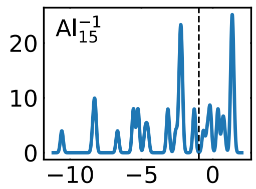

KS degeneracies within 2.72e-03 [eV]:
[1, 1, 2, 1, 2, 2, 1, 1, 2, 1, 2, 1, 1, 2, 1, 2]
KS degeneracies within 2.72e-01 [eV]:
[1, 3, 1, 2, 2, 2, 2, 8, 2]

Homo-Lumo gap: 0.64 [eV]

KS eigenvalues e_i (e_i - e_{i-1}) [eV]
-10.58791
-8.39102 (2.19689)
-8.25725 (0.13378)
-8.25721 (4.06492e-05)
-6.68034 (1.57687)
-5.56015 (1.12019)
-5.56012 (2.72626e-05)
-5.24534 (0.31478)
-5.24530 (3.91895e-05)
-4.73293 (0.51237)
-4.56022 (0.17272)
-3.14494 (1.41528)
-3.14491 (2.72303e-05)
-2.58594 (0.55897)
-2.31841 (0.26754)
-2.31838 (2.43290e-05)
-2.29333 (0.02505)
-2.22026 (0.07307)
-2.19409 (0.02617)
-2.19406 (2.74038e-05)
-2.16170 (0.03236)
-1.29719 (0.86451)
-1.29716 (2.86822e-05)

C_s

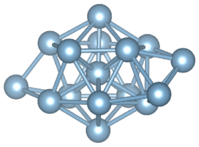

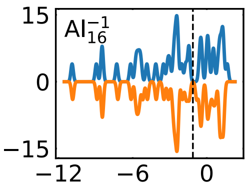

KS degeneracies within 2.72e-03 [eV]:
[[all ones, [1, 1, 2, all ones]]
KS degeneracies within 2.72e-01 [eV]:
[[1, 1, 2, 1, 1, 4, 1, 1, 1, 2, 6, 1, 3],
[1, 1, 2, 1, 1, 3, 1, 1, 1, 1, 1, 7, 1, 2]]

Homo-Lumo gap: 0.63 [eV]

KS eigenvalues e_i (e_i - e_{i-1}) [eV]
-10.90933
-8.94633 (1.96300)
-8.48606 (0.46027)
-8.47034 (0.01572)
-7.29203 (1.17832)
-6.16746 (1.12456)
-5.75276 (0.41471)
-5.61318 (0.13958)
-5.47150 (0.14168)
-5.33737 (0.13412)
-4.84975 (0.48762)
-4.12744 (0.72231)
-3.50874 (0.61871)
-3.16191 (0.34683)
-3.00378 (0.15813)
-2.67809 (0.32569)
-2.59513 (0.08297)
-2.50311 (0.09202)
-2.43126 (0.07185)
-2.37324 (0.05802)
-2.36213 (0.01111)
-2.03565 (0.32648)
-1.70154 (0.33411)
-1.45775 (0.24379)
-1.43642 (0.02133)

D_{2h}

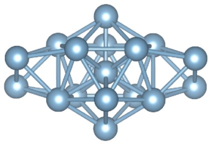

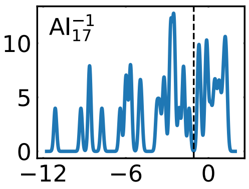

KS degeneracies within
2.72e-03 [eV]:
[all ones]
KS degeneracies within
2.72e-01 [eV]:
[1, 1, 2, 1, 1, 4, 2, 4, 6, 1, 2, 1]

Homo-Lumo gap: 0.67 [eV]

KS eigenvalues e_i (e_i - e_{i-1}) [eV]
-11.12525
-9.26042 (1.86483)
-8.64320 (0.61722)
-8.61838 (0.02482)
-7.73778 (0.88060)
-6.40346 (1.33431)
-6.03187 (0.37160)
-5.93017 (0.10169)
-5.66947 (0.26070)
-5.64519 (0.02428)
-5.00257 (0.64262)
-4.88171 (0.12086)
-3.74311 (1.13861)
-3.54588 (0.19723)
-3.27904 (0.26684)
-3.16883 (0.11020)
-2.79135 (0.37748)
-2.72599 (0.06536)
-2.70785 (0.01814)
-2.53760 (0.17025)
-2.48867 (0.04893)
-2.48459 (0.00408)
-2.13106 (0.35353)
-1.82845 (0.30262)
-1.79228 (0.03617)
-1.40295 (0.38933)

Figure continued on next page ...

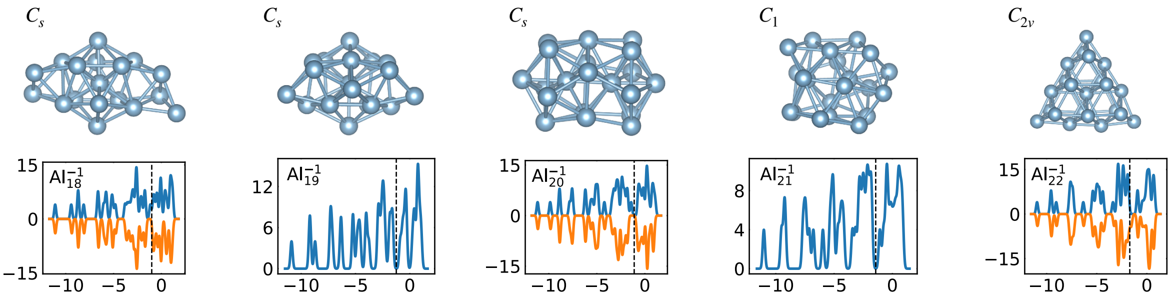

KS degeneracies within 2.72e-03 [eV]:
[[all ones], [all ones, 2, 1, 1, 1, 1]]
KS degeneracies within 2.72e-01 [eV]:
[[1, 1, 2, 1, 2, 3, 2, 1, 10, 2, 2, 1],
[1, 1, 2, 1, 2, 3, 2, 1, 2, 8, 4]]

Homo-Lumo gap: 0.30 [eV]

KS eigenvalues e_i (e_i - e_{i-1}) [eV]
-11.10390
-9.42218 (1.68172)
-8.70610 (0.71607)
-8.69681 (0.00929)
-8.12526 (0.57155)
-6.65056 (1.47470)
-6.59485 (0.05571)
-6.04729 (0.54756)
-5.81768 (0.22962)
-5.74192 (0.07575)
-5.21344 (0.52848)
-5.09779 (0.11565)
-4.81367 (0.28412)
-3.80529 (1.00838)
-3.56955 (0.23574)
-3.35340 (0.21615)
-3.13535 (0.21805)
-2.99182 (0.14353)
-2.90539 (0.08643)
-2.66142 (0.24397)
-2.64778 (0.01364)
-2.58067 (0.06711)
-2.54722 (0.03344)
-2.18846 (0.35876)
-2.05130 (0.13717)
-1.73863 (0.31267)
-1.72037 (0.01827)
-1.15742 (0.56294)

KS degeneracies within 2.72e-03 [eV]:
[all ones, 2, all ones]
KS degeneracies within 2.72e-01 [eV]:
[1, 2, 1, 3, 2, 2, 3, 3, 8, 4]

Homo-Lumo gap: 0.71 [eV]

KS eigenvalues e_i (e_i - e_{i-1}) [eV]
-10.88907
-9.22199 (1.66708)
-9.17566 (0.04633)
-8.72073 (0.45493)
-7.30570 (1.41503)
-7.29600 (0.00970)
-7.13176 (0.16424)
-6.36740 (0.76436)
-6.30147 (0.06593)
-5.23825 (1.06321)
-5.22687 (0.01139)
-4.85026 (0.37661)
-4.69075 (0.15951)
-4.57187 (0.11888)
-4.01918 (0.55269)
-4.01765 (0.00153)
-3.77022 (0.24743)
-2.91107 (0.85915)
-2.90723 (0.00384)
-2.75354 (0.15369)
-2.60330 (0.15024)
-2.51940 (0.08390)
-2.44910 (0.07030)
-2.39604 (0.05305)
-2.28252 (0.11352)
-1.86991 (0.41261)
-1.80304 (0.06687)
-1.60836 (0.19468)
-1.60080 (0.00756)

KS degeneracies within 2.72e-03 [eV]:
[[all ones], [all ones, 2, 1, 1, 1, 1, 1, 1, 1, 1]]
KS degeneracies within 2.72e-01 [eV]:
[[1, 1, 2, 3, 2, 2, 4, 1, 7, 7, 1],
[1, 1, 2, 3, 2, 1, 5, 1, 2, 5, 7]]

Homo-Lumo gap: 0.63 [eV]

KS eigenvalues e_i (e_i - e_{i-1}) [eV]
-11.16638
-9.85467 (1.31171)
-8.88374 (0.97092)
-8.87982 (0.00393)
-7.83844 (1.04138)
-7.57750 (0.26094)
-7.56082 (0.01668)
-6.21896 (1.34186)
-6.16773 (0.05122)
-5.63253 (0.53520)
-5.43467 (0.19787)
-5.13020 (0.30446)
-5.11142 (0.01879)
-4.91573 (0.19569)
-4.90999 (0.00573)
-4.21571 (0.69428)
-3.42051 (0.79520)
-3.24822 (0.17229)
-3.16445 (0.08376)
-2.95804 (0.20641)
-2.95427 (0.00377)
-2.78223 (0.17205)
-2.77085 (0.01137)
-2.43294 (0.33792)
-2.38940 (0.04354)
-2.33376 (0.05563)
-2.16950 (0.16426)
-2.07946 (0.09005)
-1.83775 (0.24171)
-1.82535 (0.01240)
-1.42460 (0.40075)

KS degeneracies within 2.72e-03 [eV]:
[all ones]
KS degeneracies within 2.72e-01 [eV]:
[1, 3, 4, 1, 1, 4, 4, 14]

Homo-Lumo gap: 0.71 [eV]

KS eigenvalues e_i (e_i - e_{i-1}) [eV]
-11.02509
-9.51115 (1.51394)
-9.28427 (0.22687)
-9.19425 (0.09002)
-7.66802 (1.52624)
-7.51901 (0.14901)
-7.39934 (0.11967)
-7.13592 (0.26342)
-6.77909 (0.35683)
-5.96193 (0.81716)
-5.35462 (0.60731)
-5.22390 (0.13072)
-5.08826 (0.13564)
-5.05817 (0.03009)
-4.67611 (0.38207)
-4.46666 (0.20945)
-4.32720 (0.13946)
-4.18505 (0.14215)
-3.42573 (0.75932)
-3.16521 (0.26052)
-2.90694 (0.25827)
-2.81997 (0.08697)
-2.72503 (0.09494)
-2.56264 (0.16239)
-2.49440 (0.06824)
-2.38855 (0.10584)
-2.21776 (0.17079)
-2.15905 (0.05871)
-2.05826 (0.10080)
-1.95409 (0.10416)
-1.82310 (0.13100)
-1.78693 (0.03617)

KS degeneracies within
2.72e-03 [eV]:
[[all ones],
[all ones, 2, 1, 1, 1, 1, 1, 2, all ones]]
KS degeneracies within
2.72e-01 [eV]:
[[1, 2, 1, 5, 1, 7, 2, 1, 5, 9],
[1, 2, 1, 5, 1, 7, 2, 1, 5, 8]]

Homo-Lumo gap: 0.29 [eV]

KS eigenvalues e_i (e_i - e_{i-1}) [eV]
-10.95944
-9.57351 (1.38593)
-9.55424 (0.01927)
-9.04808 (0.50616)
-7.71894 (1.32915)
-7.62502 (0.09392)
-7.58466 (0.04036)
-7.41060 (0.17406)
-7.38528 (0.02531)
-6.34531 (1.03997)
-5.41608 (0.92924)
-5.36559 (0.05049)
-5.11069 (0.25490)
-4.97099 (0.13970)
-4.95599 (0.01499)
-4.76045 (0.19554)
-4.74627 (0.01419)
-4.43099 (0.31527)
-4.37151 (0.05948)
-3.38343 (0.98808)
-2.94577 (0.43766)
-2.89396 (0.05181)
-2.86273 (0.03123)
-2.83527 (0.02745)
-2.76411 (0.07117)
-2.40945 (0.35466)
-2.39064 (0.01880)
-2.37711 (0.01353)
-2.31233 (0.06478)
-2.22156 (0.09077)
-1.98268 (0.23888)
-1.96631 (0.01637)
-1.86097 (0.10534)
-1.84981 (0.01116)

FIG. S3. Structures and degeneracies of the series of anionic Al clusters.

D_{3h}

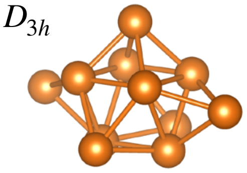

min0001 (GM)

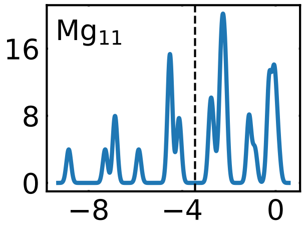

KS degeneracies within 2.72e-03 [eV]:
[1, 1, 2, 1, 2, 2, 1, 1]
KS degeneracies within 2.72e-01 [eV]:
[1, 1, 2, 1, 4, 2]

Homo-Lumo gap: 1.32 [eV]

KS eigenvalues e_i (e_i - e_{i-1}) [eV]
-8.85455
-7.29491 (1.55964)
-6.87417 (0.42074)
-6.87415 (1.72005e-05)
-5.86012 (1.01404)
-4.55165 (1.30847)
-4.55162 (2.68130e-05)
-4.49597 (0.05565)
-4.49597 (8.66982e-06)
-4.16104 (0.33492)
-4.10920 (0.05184)

C_s

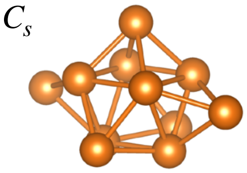

$\delta = 0.01$Å

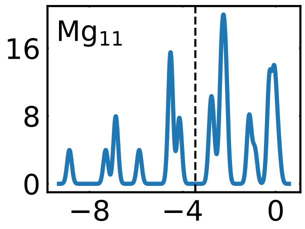

KS degeneracies within 2.72e-03 [eV]:
[1, 1, 1, 1, 1, 1, 1, 1, 1, 1, 1]
KS degeneracies within 2.72e-01 [eV]:
[1, 1, 2, 1, 4, 2]

Homo-Lumo gap: 1.32 [eV]

KS eigenvalues e_i (e_i - e_{i-1}) [eV]
-8.86900
-7.30805 (1.56096)
-6.88060 (0.42745)
-6.87194 (0.00866)
-5.86827 (1.00367)
-4.54841 (1.31986)
-4.54000 (0.00841)
-4.50173 (0.03827)
-4.49340 (0.00832)
-4.16305 (0.33035)
-4.11745 (0.04560)

C_1

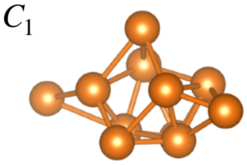

$\delta = 0.5$Å

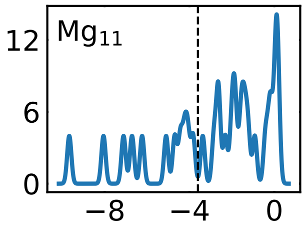

KS degeneracies within 2.72e-03 [eV]:
[1, 1, 1, 1, 1, 1, 1, 1, 1, 1, 1]
KS degeneracies within 2.72e-01 [eV]:
[1, 1, 1, 1, 1, 1, 5]

Homo-Lumo gap: 0.46 [eV]

KS eigenvalues e_i (e_i - e_{i-1}) [eV]
-9.66752
-8.05320 (1.61432)
-7.10796 (0.94524)
-6.70894 (0.39902)
-6.23332 (0.47561)
-5.10134 (1.13198)
-4.69412 (0.40723)
-4.42798 (0.26614)
-4.23038 (0.19760)
-4.07493 (0.15545)
-3.83185 (0.24308)

Figure continued on next page ...

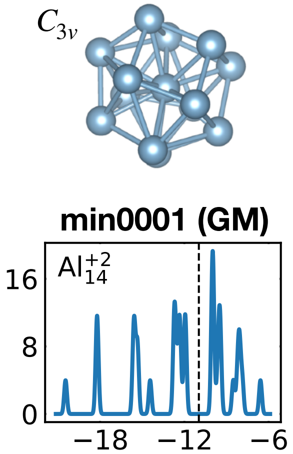

KS degeneracies within 2.72e-03 [eV]:
[1, 2, 1, 2, 1, 2, 1, 1, 2, 1, 2, 1, 1, 2]
KS degeneracies within 2.72e-01 [eV]:
[1, 3, 5, 1, 7, 3]

Homo-Lumo gap: 1.92 [eV]

KS eigenvalues e_i (e_i - e_{i-1}) [eV]
-20.45841
-18.22946 (2.22895)
-18.22940 (6.60547e-05)
-18.17873 (0.05066)
-15.60630 (2.57243)
-15.60608 (0.00023)
-15.52758 (0.07850)
-15.33591 (0.19167)
-15.33561 (0.00030)
-14.43568 (0.89993)
-12.72299 (1.71269)
-12.70054 (0.02245)
-12.70036 (0.00017)
-12.55302 (0.14734)
-12.36136 (0.19166)
-12.36121 (0.00015)
-12.28822 (0.07299)
-11.96542 (0.32280)
-11.93014 (0.03528)
-11.92992 (0.00022)

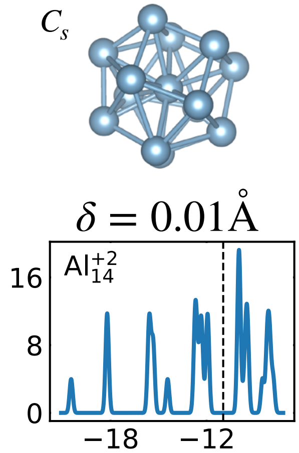

KS degeneracies within 2.72e-03 [eV]:
[1, 1, 1, 1, 1, 1, 1, 1, 1, 1, 1, 1, 1, 1, 1, 1, 1, 1, 1, 1]
KS degeneracies within 2.72e-01 [eV]:
[1, 3, 5, 1, 7, 3]

Homo-Lumo gap: 1.92 [eV]

KS eigenvalues e_i (e_i - e_{i-1}) [eV]
-20.46181
-18.23038 (2.23143)
-18.21997 (0.01041)
-18.18556 (0.03441)
-15.60806 (2.57750)
-15.59854 (0.00953)
-15.52797 (0.07056)
-15.34617 (0.18180)
-15.32921 (0.01697)
-14.43652 (0.89269)
-12.72528 (1.71123)
-12.70442 (0.02086)
-12.69129 (0.01313)
-12.55545 (0.13584)
-12.37068 (0.18476)
-12.35491 (0.01578)
-12.27920 (0.07570)
-11.97332 (0.30588)
-11.93066 (0.04266)
-11.92439 (0.00627)

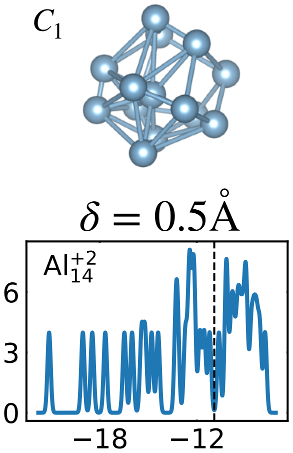

KS degeneracies within 2.72e-03 [eV]:
[1, 1, 1, 1, 1, 1, 1, 1, 1, 1, 1, 1, 1, 1, 1, 1, 1, 1, 1, 1]
KS degeneracies within 2.72e-01 [eV]:
[1, 1, 1, 1, 1, 1, 2, 1, 1, 2, 5, 2, 1]

Homo-Lumo gap: 0.57 [eV]

KS eigenvalues e_i (e_i - e_{i-1}) [eV]
-21.12539
-19.04121 (2.08418)
-18.45860 (0.58261)
-17.65299 (0.80561)
-16.47132 (1.18167)
-15.99913 (0.47219)
-15.41084 (0.58829)
-15.19507 (0.21577)
-14.79044 (0.40464)
-14.38801 (0.40243)
-13.30152 (1.08648)
-13.18047 (0.12105)
-12.70541 (0.47506)
-12.47920 (0.22621)
-12.41180 (0.06740)
-12.22514 (0.18667)
-12.14920 (0.07594)
-11.80942 (0.33978)
-11.54083 (0.26860)
-11.20645 (0.33438)

Figure continued on next page ...

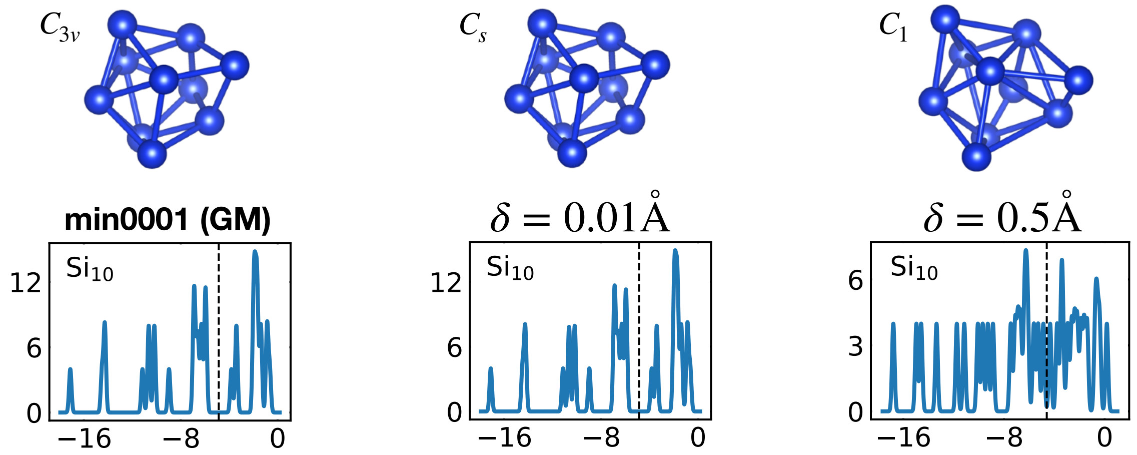

KS degeneracies within 2.72e-03 [eV]:
[1, 1, 2, 1, 2, 2, 1, 1, 2, 1, 1, 2, 2, 1]
KS degeneracies within 2.72e-01 [eV]:
[1, 3, 1, 2, 2, 1, 7, 3]

Homo-Lumo gap: 2.09 [eV]

KS eigenvalues e_i (e_i - e_{i-1}) [eV]
-17.12166
-14.50120 (2.62046)
-14.27155 (0.22965)
-14.27140 (0.00015)
-11.16173 (3.10966)
-10.64567 (0.51606)
-10.64546 (0.00021)
-10.18284 (0.46262)
-10.18276 (8.24397e-05)
-8.97187 (1.21088)
-6.94153 (2.03035)
-6.87553 (0.06600)
-6.87535 (0.00018)
-6.65232 (0.22303)
-6.56035 (0.09197)
-6.29172 (0.26863)
-6.29156 (0.00015)
-5.97521 (0.31636)
-5.97510 (0.00011)
-5.91293 (0.06217)

KS degeneracies within 2.72e-03 [eV]:
[1, 1, 1, 1, 1, 1, 1, 1, 1, 1, 1, 1, 1, 1, 1, 1,
1, 1, 1, 1, 1]
KS degeneracies within 2.72e-01 [eV]:
[1, 3, 1, 2, 2, 1, 7, 3]

Homo-Lumo gap: 2.08 [eV]

KS eigenvalues e_i (e_i - e_{i-1}) [eV]
-17.10782
-14.49016 (2.61765)
-14.28971 (0.20045)
-14.23991 (0.04980)
-11.14278 (3.09713)
-10.66491 (0.47787)
-10.62589 (0.03902)
-10.18913 (0.43675)
-10.16849 (0.02064)
-8.97303 (1.19546)
-6.93467 (2.03836)
-6.88416 (0.05051)
-6.85779 (0.02637)
-6.65583 (0.20196)
-6.55913 (0.09670)
-6.29931 (0.25982)
-6.28201 (0.01729)
-5.98017 (0.30185)
-5.97339 (0.00678)
-5.90297 (0.07042)

KS degeneracies within 2.72e-03 [eV]:
[1, 1, 1, 1, 1, 1, 1, 1, 1, 1, 1, 1, 1, 1, 1,
1, 1, 1, 1, 1]
KS degeneracies within 2.72e-01 [eV]:
[1, 1, 1, 1, 1, 1, 1, 1, 1, 1, 1, 3, 3, 1, 1, 1]

Homo-Lumo gap: 0.54 [eV]

KS eigenvalues e_i (e_i - e_{i-1}) [eV]
-16.79156
-14.95667 (1.83489)
-14.58116 (0.37551)
-13.35817 (1.22299)
-11.74541 (1.61276)
-11.15529 (0.59012)
-10.07269 (1.08259)
-9.69380 (0.37889)
-9.28600 (0.40780)
-8.87694 (0.40906)
-7.46001 (1.41693)
-7.11702 (0.34299)
-6.89036 (0.22666)
-6.68185 (0.20851)
-6.37897 (0.30288)
-6.25232 (0.12665)
-6.11478 (0.13753)
-5.62673 (0.48805)
-5.28749 (0.33925)
-4.87315 (0.41433)

FIG. S4. Lifting of the KS eigenvalue degeneracies by random atomic displacements away from the global minima. The effect for two different displacement amplitudes δ is shown, $\delta = 0.01$ and 0.5 Å.

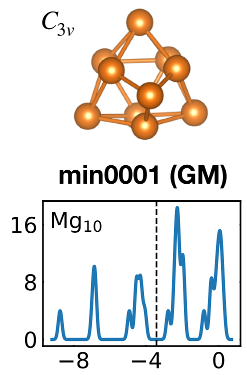

$E_{KS} - E^0_{KS}$: 0.000 [eV]
Surface/Volume ratio: 0.62191
Shape factor: 0.07
Average coordination: 5.52
Homo-Lumo gap: 1.29 [eV]
Chemical Hardness: 2.11 [eV]

Point group degeneracies: [1, 2]
KS eigenvalues degeneracies:
[1, 1, 2, 1, 2, 2, 1]

KS eigenvalues e_i (e_i - e_{i-1}) [eV]
-8.74596
-6.95134 (1.79461)
-6.82749 (0.12385)
-6.82744 (5.35252e-05)
-4.92729 (1.90014)
-4.53743 (0.38986)
-4.53737 (5.94869e-05)
-4.31307 (0.22430)
-4.31299 (8.17428e-05)
-4.07739 (0.23559)

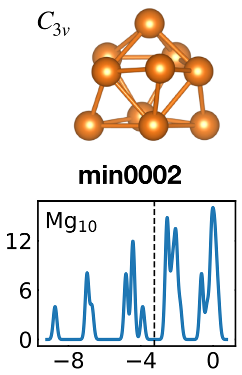

$E_{KS} - E^0_{KS}$: 0.076 [eV]
Surface/Volume ratio: 0.62520
Shape factor: -0.09
Average coordination: 5.61
Homo-Lumo gap: 1.29 [eV]
Chemical Hardness: 2.11 [eV]

Point group degeneracies: [1, 2]
KS eigenvalues degeneracies:
[1, 2, 1, 2, 1, 2, 1]

KS eigenvalues e_i (e_i - e_{i-1}) [eV]
-8.73841
-6.96839 (1.77001)
-6.96835 (3.71656e-05)
-6.70153 (0.26682)
-4.81927 (1.88226)
-4.81897 (0.00030)
-4.44410 (0.37486)
-4.43125 (0.01285)
-4.43115 (9.87531e-05)
-3.89355 (0.53760)

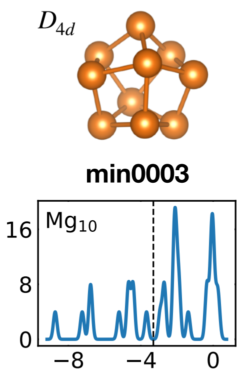

$E_{KS} - E^0_{KS}$: 0.254 [eV]
Surface/Volume ratio: 0.61777
Shape factor: 0.15
Average coordination: 5.62
Homo-Lumo gap: 0.75 [eV]
Chemical Hardness: 1.86 [eV]

Point group degeneracies: [1, 2]
KS eigenvalues degeneracies:
[1,1,2,1,2,2,1]

KS eigenvalues e_i (e_i - e_{i-1}) [eV]
-8.72873
-7.23557 (1.49315)
-6.77194 (0.46362)
-6.77192 (2.55979e-05)
-5.19954 (1.57237)
-4.67866 (0.52087)
-4.67863 (3.57179e-05)
-4.43095 (0.24767)
-4.42893 (0.00201)
-3.68070 (0.74822)

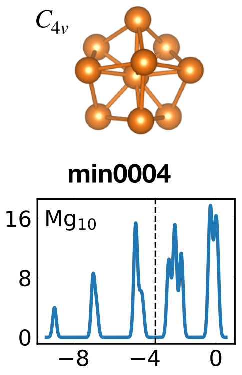

$E_{KS} - E^0_{KS}$: 0.600 [eV]
Surface/Volume ratio: 0.64243
Shape factor: -0.03
Average coordination: 5.84
Homo-Lumo gap: 1.44 [eV]
Chemical Hardness: 2.16 [eV]

Point group degeneracies: [1, 2]
KS eigenvalues degeneracies:
1,2,1,1,2,1,1,1]

KS eigenvalues e_i (e_i - e_{i-1}) [eV]
-9.06310
-6.90100 (2.16210)
-6.90100 (1.43087e-06)
-6.70193 (0.19906)
-4.55902 (2.14290)
-4.49958 (0.05944)
-4.49957 (7.29703e-06)
-4.47042 (0.02915)
-4.26262 (0.20779)
-4.11483 (0.14779)

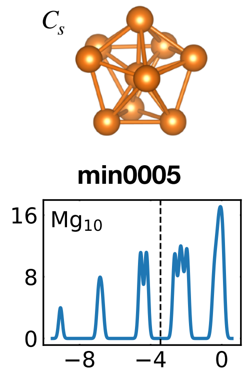

$E_{KS} - E^0_{KS}$: 0.604 [eV]
Surface/Volume ratio: 0.64145
Shape factor: 0.01
Average coordination: 5.83
Homo-Lumo gap: 1.47 [eV]
Chemical Hardness: 2.18 [eV]

Point group degeneracies: [1]
KS eigenvalues degeneracies: [all 1]

KS eigenvalues e_i (e_i - e_{i-1}) [eV]
-9.07503
-6.95491 (2.12011)
-6.84213 (0.11277)
-6.71843 (0.12369)
-4.62017 (2.09825)
-4.55214 (0.06803)
-4.52419 (0.02794)
-4.27916 (0.24502)
-4.24782 (0.03134)
-4.18317 (0.06464)

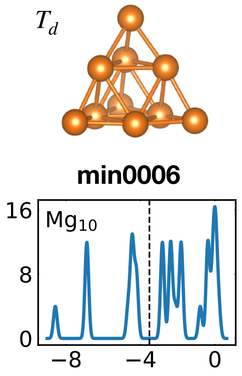

$E_{KS} - E^0_{KS}$: 0.644 [eV]
Surface/Volume ratio: 0.64658
Shape factor: 0.00
Average coordination: 4.99
Homo-Lumo gap: 1.40 [eV]
Chemical Hardness: 2.09 [eV]

Point group degeneracies: [1, 2, 3]
KS eigenvalues degeneracies: [1,3,1,3,2]

KS eigenvalues e_i (e_i - e_{i-1}) [eV]
-8.56985
-6.87731 (1.69253)
-6.87730 (8.22007e-06)
-6.87727 (3.10549e-05)
-4.65333 (2.22394)
-4.44196 (0.21136)
-4.44193 (3.28330e-05)
-4.44189 (3.88585e-05)
-4.21442 (0.22747)
-4.21438 (3.42474e-05)

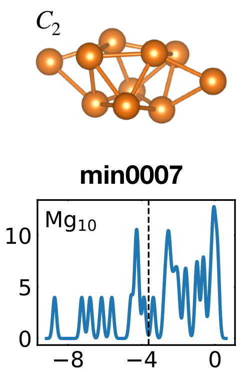

$E_{KS} - E^0_{KS}$: 1.005 [eV]
Surface/Volume ratio: 0.65107
Shape factor: 0.24
Average coordination: 5.37
Homo-Lumo gap: 0.51 [eV]
Chemical Hardness: 1.73 [eV]

Point group degeneracies: [1]
KS eigenvalues degeneracies: [all 1]

KS eigenvalues e_i (e_i - e_{i-1}) [eV]
-8.73716
-7.25752 (1.47963)
-6.80521 (0.45231)
-6.17887 (0.62633)
-5.62209 (0.55678)
-4.57221 (1.04987)
-4.31913 (0.25308)
-4.27806 (0.04106)
-4.19585 (0.08220)
-3.87341 (0.32244)

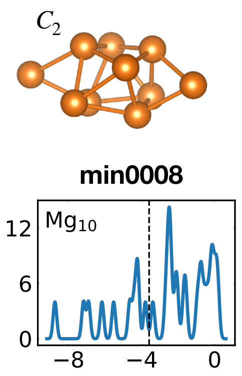

$E_{KS} - E^0_{KS}$: 1.016 [eV]
Surface/Volume ratio: 0.65400
Shape factor: 0.21
Average coordination: 5.42
Homo-Lumo gap: 0.41 [eV]
Chemical Hardness: 1.71 [eV]

Point group degeneracies: [1]
KS eigenvalues degeneracies: [all 1]

KS eigenvalues e_i (e_i - e_{i-1}) [eV]
-8.73562
-7.17074 (1.56488)
-6.89645 (0.27429)
-6.16269 (0.73377)
-5.52272 (0.63996)
-4.64261 (0.88012)
-4.40931 (0.23330)
-4.21977 (0.18953)
-4.21257 (0.00720)
-3.78663 (0.42594)

FIG. S5. Structural and electronic properties of the first eight low-lying minima for the Mg_{10} cluster.

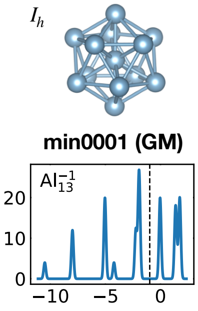

$E_{KS} - E_{KS}^0$: 0.000 [eV]
Surface/Volume ratio: 0.35506
Shape factor: 0.00
Average coordination: 6.46
Homo-Lumo gap: 1.88 [eV]
Chemical Hardness: 2.52 [eV]

Point group degeneracies: [1, 3, 4, 5]
KS eigenvalues degeneracies: [1, 3, 5, 1, 3

KS eigenvalues e_i (e_i - e_{i-1}) [eV]
-10.49679
-7.98559 (2.51120)
-7.98558 (9.89332e-06)
-7.98558 (5.17805e-06)
-5.03997 (2.94560)
-5.03997 (9.44743e-06)
-5.03996 (9.24497e-06)
-5.03995 (6.27813e-06)
-5.03994 (8.49797e-06)
-4.21843 (0.82151)
-2.24053 (1.97791)
-2.24052 (3.08380e-06)
-2.24052 (6.61844e-06)
-1.97855 (0.26196)
-1.97855 (7.13253e-06)
-1.97854 (8.45329e-06)
-1.91787 (0.06066)
-1.91786 (0.00001)
-1.91786 (5.29521e-06)
-1.91785 (3.71857e-06)

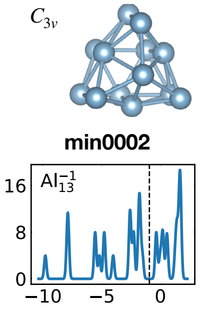

$E_{KS} - E_{KS}^0$: 0.971 [eV]
Surface/Volume ratio: 0.35709
Shape factor: 0.02
Average coordination: 5.47
Homo-Lumo gap: 1.18 [eV]
Chemical Hardness: 2.07 [eV]

Point group degeneracies: [1, 2]
KS eigen. deg: [1, 1, 2, 2, 1, 2, 1, 2, 1, 2, 1,

KS eigenvalues e_i (e_i - e_{i-1}) [eV]
-9.77744
-7.91554 (1.86190)
-7.84541 (0.07013)
-7.84524 (0.00017)
-5.54248 (2.30276)
-5.54246 (2.65423e-05)
-5.18395 (0.35851)
-4.77112 (0.41283)
-4.77094 (0.00018)
-4.01450 (0.75643)
-2.59232 (1.42219)
-2.59228 (3.14752e-05)
-2.55009 (0.04219)
-2.27421 (0.27589)
-2.27415 (5.24515e-05)
-1.86472 (0.40943)
-1.77609 (0.08863)
-1.75675 (0.01934)
-1.75651 (0.00024)
-1.52764 (0.22887)

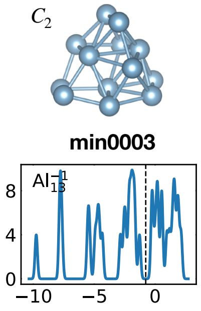

$E_{KS} - E_{KS}^0$: 1.474 [eV]
Surface/Volume ratio: 0.36061
Shape factor: 0.06
Average coordination: 5.44
Homo-Lumo gap: 1.02 [eV]
Chemical Hardness: 1.98 [eV]

Point group degeneracies: [1]
KS eigenvalues degeneracies: [1, 2, all 1]

KS eigenvalues e_i (e_i - e_{i-1}) [eV]
-9.75384
-7.79719 (1.95664)
-7.79628 (0.00092)
-7.65658 (0.13970)
-5.52057 (2.13601)
-5.39940 (0.12117)
-4.91018 (0.48922)
-4.71381 (0.19637)
-4.58975 (0.12406)
-4.33400 (0.25575)
-2.84425 (1.48975)
-2.55442 (0.28983)
-2.42715 (0.12726)
-2.15626 (0.27090)
-2.08679 (0.06946)
-1.93860 (0.14819)
-1.87976 (0.05884)
-1.74098 (0.13878)
-1.67012 (0.07086)
-1.28025 (0.38987)

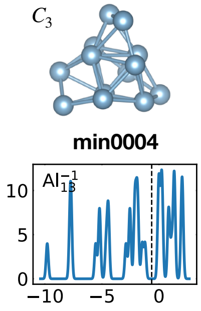

$E_{KS} - E_{KS}^0$: 1.541 [eV]
Surface/Volume ratio: 0.36301
Shape factor: -0.05
Average coordination: 5.47
Homo-Lumo gap: 1.16 [eV]
Chemical Hardness: 2.05 [eV]

Point group degeneracies: [1, 2]
KS eigenvalues degeneracies: [1, 2, all 1]

KS eigenvalues e_i (e_i - e_{i-1}) [eV]
-9.78831
-7.79987 (1.98844)
-7.71464 (0.08523)
-7.71450 (0.00014)
-5.54567 (2.16883)
-5.20982 (0.33585)
-5.20957 (0.00025)
-4.62556 (0.58401)
-4.44087 (0.18469)
-4.44044 (0.00042)
-2.89109 (1.54935)
-2.54841 (0.34268)
-2.54812 (0.00028)
-2.11739 (0.43073)
-2.11728 (0.00011)
-1.98402 (0.13326)
-1.86917 (0.11485)
-1.86892 (0.00025)
-1.47482 (0.39410)
-1.22195 (0.25287)

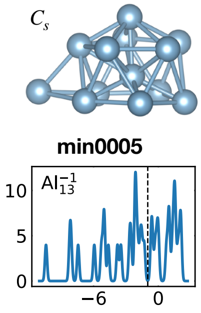

$E_{KS} - E_{KS}^0$: 1.828 [eV]
Surface/Volume ratio: 0.36129
Shape factor: 0.11
Average coordination: 5.99
Homo-Lumo gap: 0.65 [eV]
Chemical Hardness: 1.86 [eV]

Point group degeneracies: [1]
KS eigenvalues degeneracies: [all 1]

KS eigenvalues e_i (e_i - e_{i-1}) [eV]
-10.40753
-8.20539 (2.20214)
-8.08868 (0.11671)
-7.43199 (0.65668)
-5.93330 (1.49869)
-5.26603 (0.66727)
-5.05272 (0.21331)
-4.99841 (0.05431)
-4.63033 (0.36809)
-3.78496 (0.84537)
-3.47023 (0.31473)
-2.66203 (0.80820)
-2.52978 (0.13224)
-2.14485 (0.38493)
-2.13533 (0.00952)
-2.08966 (0.04567)
-1.90622 (0.18343)
-1.67158 (0.23465)
-1.52621 (0.14536)
-1.30600 (0.22022)

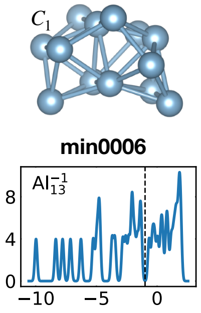

$E_{KS} - E_{KS}^0$: 1.864 [eV]
Surface/Volume ratio: 0.36213
Shape factor: 0.09
Average coordination: 5.55
Homo-Lumo gap: 0.67 [eV]
Chemical Hardness: 1.81 [eV]

Point group degeneracies: [1]
KS eigenvalues degeneracies: [all 1]

KS eigenvalues e_i (e_i - e_{i-1}) [eV]
-9.97308
-8.34336 (1.62972)
-7.78367 (0.55969)
-7.13623 (0.64744)
-6.27397 (0.86226)
-5.26156 (1.01240)
-4.98251 (0.27905)
-4.80379 (0.17872)
-4.72911 (0.07467)
-3.70980 (1.01932)
-3.47887 (0.23093)
-2.83001 (0.64886)
-2.58325 (0.24676)
-2.32931 (0.25394)
-2.07735 (0.25196)
-2.05402 (0.02333)
-1.84897 (0.20505)
-1.61974 (0.22923)
-1.40131 (0.21844)
-1.32678 (0.07453)

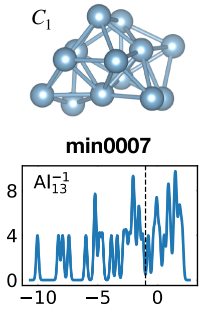

$E_{KS} - E_{KS}^0$: 1.869 [eV]
Surface/Volume ratio: 0.36236
Shape factor: 0.09
Average coordination: 5.67
Homo-Lumo gap: 0.47 [eV]
Chemical Hardness: 1.71 [eV]

Point group degeneracies: [1]
KS eigenvalues degeneracies: [all 1]

KS eigenvalues e_i (e_i - e_{i-1}) [eV]
-10.03787
-8.28153 (1.75634)
-7.93435 (0.34718)
-7.40839 (0.52596)
-5.98557 (1.42282)
-5.24638 (0.73919)
-5.19363 (0.05275)
-4.85452 (0.33911)
-4.61292 (0.24161)
-3.85762 (0.75529)
-3.36388 (0.49375)
-2.82409 (0.53978)
-2.60609 (0.21801)
-2.33636 (0.26973)
-2.06964 (0.26672)
-2.05645 (0.01319)
-1.90003 (0.15641)
-1.61889 (0.28114)
-1.49520 (0.12370)
-1.23781 (0.25738)

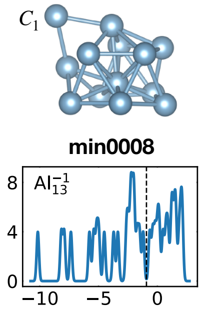

$E_{KS} - E_{KS}^0$: 1.891 [eV]
Surface/Volume ratio: 0.36146
Shape factor: 0.01
Average coordination: 5.78
Homo-Lumo gap: 0.56 [eV]
Chemical Hardness: 1.77 [eV]

Point group degeneracies: [1]
KS eigenvalues degeneracies: [all 1]

KS eigenvalues e_i (e_i - e_{i-1}) [eV]
-10.15829
-8.16150 (1.99678)
-7.91758 (0.24392)
-7.34792 (0.56966)
-5.77109 (1.57683)
-5.41241 (0.35867)
-5.16911 (0.24331)
-4.98082 (0.18829)
-4.51313 (0.46769)
-3.71488 (0.79825)
-3.35735 (0.35752)
-2.62501 (0.73235)
-2.46654 (0.15847)
-2.25972 (0.20681)
-2.21365 (0.04607)
-2.03091 (0.18274)
-1.99038 (0.04052)
-1.72825 (0.26213)
-1.52004 (0.20821)
-1.20655 (0.31349)

FIG. S6. Structural and electronic properties of the first eight low-lying minima for the Al_{13}^{-1} cluster.

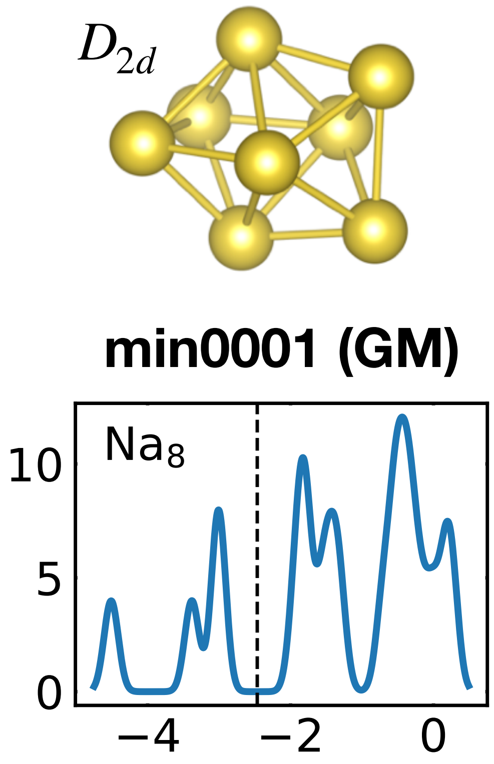

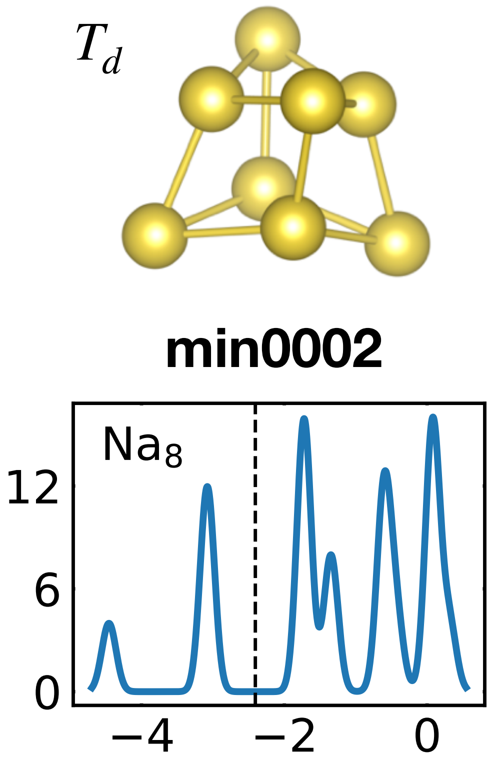

$E_{KS} - E_{KS}^0$: 0.000 [eV]
Surface/Volume ratio: 0.84330
Shape factor: 0.23
Average coordination: 4.49
Homo-Lumo gap: 1.08 [eV]
Chemical Hardness: 1.84 [eV]

Point group degeneracies: [1,2]
KS eigenvalues degeneracies: [1, 1, 2]

KS eigenvalues e_i (e_i - e_{i-1}) [eV]
-4.51033
-3.38137 (1.12895)
-3.00809 (0.37328)
-3.00765 (0.00044)

$E_{KS} - E_{KS}^0$: 0.093 [eV]
Surface/Volume ratio: 0.84345
Shape factor: 0.00
Average coordination: 4.50
Homo-Lumo gap: 1.34 [eV]
Chemical Hardness: 1.91 [eV]

Point group degeneracies: [1, 2, 3]
KS eigenvalues degeneracies: [1, 3]

KS eigenvalues e_i (e_i - e_{i-1}) [eV]
-4.45624
-3.07899 (1.37724)
-3.07825 (0.00074)
-3.07788 (0.00036)

FIG. S7. Structural and electronic properties of the first two low-lying minima for the Na_8 cluster. The two structures are completely unrelated and the degeneracy of the KS eigenvalues in the ground state is accidental and not caused by symmetry.

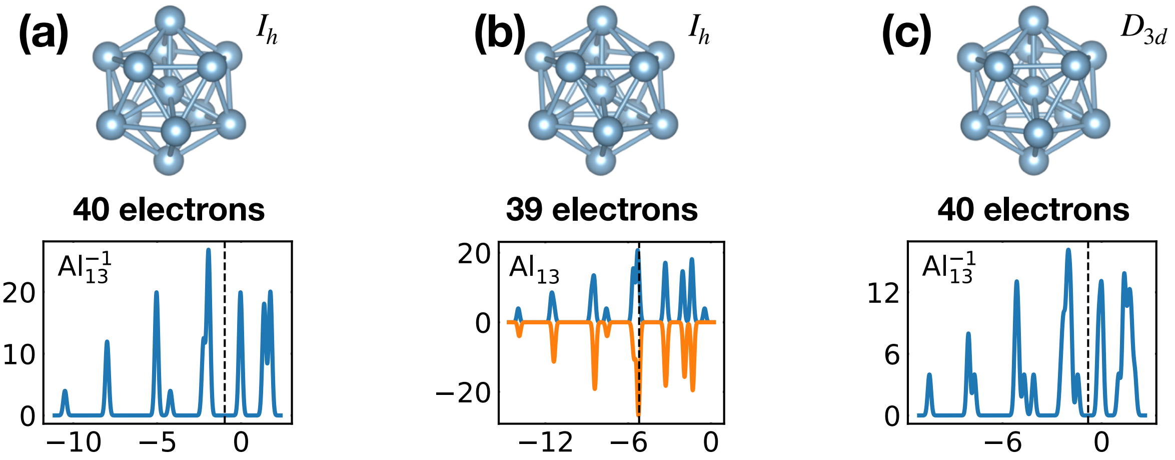

KS eigenvalues e_i (e_i - e_{i-1}) [eV]	KS eigenvalues e_i (e_i - e_{i-1}) [eV]	KS eigenvalues e_i (e_i - e_{i-1}) [eV]
-10.49679	-13.96431	-10.43408
-7.98559 (2.51120)	-11.57869 (2.38562)	-8.06992 (2.36416)
-7.98558 (9.89332e-06)	-11.52573 (0.05297)	-8.06972 (0.00020)
-7.98558 (5.17805e-06)	-11.36706 (0.15867)	-7.70984 (0.35987)
-5.03997 (2.94560)	-8.68364 (2.68341)	-5.20525 (2.50459)
-5.03997 (9.44743e-06)	-8.63234 (0.05130)	-5.20446 (0.00079)
-5.03996 (9.24497e-06)	-8.48062 (0.15172)	-5.07875 (0.12572)
-5.03995 (6.27813e-06)	-8.46478 (0.01584)	-5.07786 (0.00088)
-5.03994 (8.49797e-06)	-8.44586 (0.01892)	-4.69824 (0.37963)
-4.21843 (0.82151)	-7.57769 (0.86817)	-4.11989 (0.57835)
-2.24053 (1.97791)	-5.66563 (1.91205)	-2.45946 (1.66043)
-2.24052 (3.08380e-06)	-5.65498 (0.01066)	-2.29754 (0.16192)
-2.24052 (6.61844e-06)	-5.60219 (0.05278)	-2.29726 (0.00028)
-1.97855 (0.26196)	-5.60175 (0.00045)	-2.07816 (0.21909)
-1.97855 (7.13253e-06)	-5.36922 (0.23253)	-2.07811 (5.15796e-05)
-1.97854 (8.45329e-06)	-5.34244 (0.02678)	-2.06937 (0.00874)
-1.91787 (0.06066)	-5.32496 (0.01748)	-1.94365 (0.12573)
-1.91786 (1.13828e-05)	-5.28777 (0.03719)	-1.87401 (0.06964)
-1.91786 (5.29521e-06)	-5.28081 (0.00696)	-1.87276 (0.00125)
-1.91785 (3.71857e-06)	-5.18421 (0.09660)	-1.45387 (0.41888)

FIG. S8. Density of states of 40 (a) and 39 (b) valence electrons, distributed over a perfect icosahedron with point group I_h. This structure is the GM of Al_{13}^{-1} and has point group degeneracies [1, 3, 4, 5]. (c) shows the DoS of 40 electrons distributed over the slightly distorted icosahedron D_{3d} GM of Al_{13} which has point group degeneracies [1, 2].
